# Outer Membrane Vesicles From *Brucella melitensis* Modulate Immune Response and Induce Cytoskeleton Rearrangement in Peripheral Blood Mononuclear Cells

**DOI:** 10.3389/fmicb.2020.556795

**Published:** 2020-10-19

**Authors:** Eric Daniel Avila-Calderón, Olín Medina-Chávez, Leopoldo Flores-Romo, José Manuel Hernández-Hernández, Luis Donis-Maturano, Ahidé López-Merino, Beatriz Arellano-Reynoso, Ma. Guadalupe Aguilera-Arreola, Enrico A. Ruiz, Zulema Gomez-Lunar, Sharon Witonsky, Araceli Contreras-Rodríguez

**Affiliations:** ^1^Departamento de Microbiología, Escuela Nacional de Ciencias Biológicas, Instituto Politécnico Nacional, Mexico City, Mexico; ^2^Departamento de Biología Celular, Centro de Investigación y de Estudios Avanzados, Instituto Politécnico Nacional, Mexico City, Mexico; ^3^Centro de Biotecnología FEMSA, Escuela de Ingeniería y Ciencias, Instituto Tecnológico y de Estudios Superiores de Monterrey, Monterrey, Mexico; ^4^Unidad de Investigación en Biomedicina, Facultad de Estudios Superiores Iztacala, Universidad Nacional Autónoma de México, Mexico City, Mexico; ^5^Departamento de Microbiología e Inmunología, Facultad de Medicina Veterinaria y Zootecnia, Universidad Nacional Autónoma de México, Mexico City, Mexico; ^6^Departamento de Zoología, Escuela Nacional de Ciencias Biológicas, Instituto Politécnico Nacional, Mexico City, Mexico; ^7^Center for One Health Research, Virginia-Maryland College of Veterinary Medicine, Virginia Tech, Blacksburg, VA, United States; ^8^Large Animal Clinical Sciences, Virginia-Maryland College of Veterinary Medicine, Virginia Tech, Blacksburg, VA, United States

**Keywords:** outer membrane vesicles, *Brucella*, proteomics, OMVs, bacterial vesicles, extracellular vesicle

## Abstract

Similar to what has been described in other Gram-negative bacteria, *Brucella melitensis* releases outer membrane vesicles (OMVs). OMVs from *B. melitensis* 16M and the rough-mutant *B. melitensis* VTRM1 were able to induce a protective immune response against virulent *B. melitensis* in mice models. The presence of some proteins which had previously been reported to induce protection against *Brucella* were found in the proteome of OMVs from *B. melitensis* 16M. However, the proteome of OMVs from *B. melitensis* VTRM1 had not previously been determined. In order to be better understand the role of OMVs in host-cell interactions, the aim of this work was to compare the proteomes of OMVs from *B. melitensis* 16M and the derived rough-mutant *B. melitensis* VTRM1, as well as to characterize the immune response induced by vesicles on host cells. Additionally, the effect of SDS and proteinase K on the stability of OMVs was analyzed. OMVs from *B. melitensis* 16M (smooth strain) and the *B. melitensis* VTRM1 rough mutant (lacking the *O*-polysaccharide side chain) were analyzed through liquid chromatography-mass spectrometry (LC-MS/MS). OMVs were treated with proteinase K, sodium deoxycholate, and SDS, and then their protein profile was determined using SDS-PAGE. Furthermore, PBMCs were treated with OMVs in order to measure their effect on cytoskeleton, surface molecules, apoptosis, DNA damage, proliferation, and cytokine-induction. A total of 131 proteins were identified in OMVs from *B. melitensis*16M, and 43 in OMVs from *B. melitensis* VTRM1. Proteome comparison showed that 22 orthologous proteins were common in vesicles from both strains, and their core proteome contained Omp31, Omp25, GroL, and Omp16. After a subsequent detergent and enzyme treatment, OMVs from *B. melitensis* VTRM1 exhibited higher sensitive compared to OMVs from the *B. melitensis* 16M strain. Neither OMVs induced IL-17, proliferation, apoptosis or DNA damage. Nonetheless, OMVs from the smooth and rough strains induced overproduction of TNFα and IL-6, as well as actin and tubulin rearrangements in the cytoskeleton. Moreover, OMVs from both strains inhibited PD-L1 expression in T-cells. These data revealed significant differences in OMVs derived from the rough and smooth *Brucella* strains, among which, the presence or absence of complete LPS appeared to be crucial to protect proteins contained within vesicles and to drive the immune response.

## Introduction

*Brucella* is a Gram-negative pathogen that can cause brucellosis, a worldwide zoonotic disease still prevalent in many countries. In animals, brucellosis can lead to abortion and infertility in infected males. Consumption of unpasteurized dairy-products made using milk from infected animals is the main infection cause for humans. Initial illness symptoms include fever, sweating, malaise, headaches, backaches, joint and muscle pain. When symptoms persist for more than a year, brucellosis is then classified as chronic, developing different signs and symptoms such as arthritis, endocarditis, recurrent fevers, and depression (Seleem et al., 2010).

Similar to other Gram-negative bacteria, *B. melitensis* releases OMVs (Lynch and Alegado, 2017). These vesicles are nanostructures consisting of a bi-lipid membrane and a spherical shape, ranging from 25 to 250 nm in size. Production of OMVs begins with membrane bulging and ends with membrane fusion, followed by the release of vesicles into the external space (Toyofuku et al., 2019). Many studies have contributed in determining the composition of bacterial vesicles, which include phospholipids, outer membrane proteins (OMPs), lipopolysaccharides (LPS), periplasmic/cytoplasmic proteins, as well as nucleic acids (Pathirana and Kaparakis-Liaskos, 2016; Bitto et al., 2017).

Given that these nanostructures contain molecules from the progenitor bacterial cell, OMVs can interact with the host cells. OMVs can trigger different immune responses both *in vitro* and *in vivo*. OMVs purified from *Aeromonas hydrophila* induced the expression of the surface activation marker CD69 in B and T lymphocytes *in vitro* (Avila-Calderón et al., 2018). On the other hand, OMVs have been used as an *in vivo* vaccine, inducing protection in mice after exposing them with the virulent strain as observed in OMVs from *Acinetobacter baumannii*, *Helicobacter pylori*, *Escherichia coli*, and *Brucella* (Avila-Calderón et al., 2012; Araiza-Villanueva et al., 2019; Liu et al., 2019; Wang et al., 2019; Pulido et al., 2020).

Previous testing of OMVs purified from *B. melitensis* and *B. abortus* as an acellular vaccine in mice, resulted in a level of protection comparable to that obtained from live *Brucella* vaccines (Avila-Calderón et al., 2012; Araiza-Villanueva et al., 2019). Such results demonstrated that OMVs are not infectious unlike whole *Brucella* cells, and they elicited a cell-mediated protective immune response to kill the intracellular pathogen (Avila-Calderón et al., 2012; Araiza-Villanueva et al., 2019). Additionally, several proteins involved in the immune response against *B. abortus and B. melitensis* were found within their OMVs. Interestingly, when comparing OMVs from the *B. melitensis* and *B. abortus* rough strains against smooth strains from the same species, important differences were observed. For instance the absence of the *O*-polysaccharide side chain of the LPS in rough strains was shown to improve the exposition of protein epitopes in OMVs; thus, increasing immune protection *in vivo* (Avila-Calderón et al., 2012; Araiza-Villanueva et al., 2019). The LPS has been related to biogenesis of OMVs as well as to the selective mechanism of protein sorting (Bonnington and Kuehn, 2014; Murphy et al., 2014; Cahill et al., 2015). *Brucella*-LPS are crucial molecules that contribute to virulence, and to the stealthy nature of this facultative intracellular pathogen (Cardoso et al., 2006).

Outer membrane vesicles from *B. melitensis* 16M and the rough-mutant *B. melitensis* VTRM1 were able to elicit a protective immune response against virulent *B. melitensis* in mice models. The presence of some proteins which had previously been reported to induce protection against *Brucella* were found in the proteome of OMVs from *B. melitensis* 16M. However, the proteome of OMVs from *B. melitensis* VTRM1 had not previously been determined. For a better understanding in the role of OMVs, pertaining to host-cell interactions, the aim of the present work was to compare the proteome of OMVs from *B. melitensis* 16M and the derived rough-mutant *B. melitensis* VTRM1, as well as to characterize the immune response induced by vesicles on host cells. Furthermore, the effect of SDS and proteinase K on OMVs integrity was analyzed. In this study, OMVs from rough and smooth *B. melitensis* strains were compared to assess the effect of the LPS O-side chain on host cell response, protein composition and resistance to detergents as well as proteinase K.

## Materials and Methods

### Purification of OMVs

Purification of OMVs was performed according to the protocol described by Avila-Calderón et al. (2012). In short, the *B. melitensis* 16M and VTRM1 strains were cultured on tryptic soy agar plates supplemented with 0.5% yeast extract (TSA-YE) (Becton Dickinson-BD^TM^) and incubated 24 h at 37°C. The bacteria were harvested with a rubber policeman and suspended in 250 mL sterile PBS 0.1M (Gibco^®^). The bacterial suspension was centrifuged at 10,000 × *g* for 30 min (Thermo Scientific^TM^ Sorvall^TM^ Legend^TM^ XT/XF.). The supernatant was decanted through a 0.22 μm filter (Millipore Corp.), and a sterility test was performed by culturing an aliquot on a TSA-YE plate followed by incubation for 1 week at 37°C. The sterile supernatant was centrifuged at 100,000 × *g* for 2 h at 4°C (Beckman Coulter Optima L-90K). The pellet containing the isolated OMVs was washed twice using 25 mL sterile PBS and OMVs were suspended in 1 mL sterile PBS (OMVs). Total protein concentration was determined using the PIERCE-BCA (Thermo Fisher Scientific Inc.) reagents, following the manufacturer’s recommendations. OMVs were purified through a density-gradient with the aid of the OptiPrep protocol (Sigma-Aldrich, Inc.), following the Fernandez-Moreira et al. (2006). Concisely, sterile PBS-OptiPrep solutions were prepared at final concentrations of 10, 15, 20, 25, and 30%. A volume of 2.6 mL from these OptiPrep solutions were subsequentially layered in an ultracentrifuge tube from the highest to the lowest density. OMVs were placed at the bottom of the tube. Tubes were centrifuged at 100,000 × *g* for 16 h at 4°C. Finally, OMVs could be observed as an opalescent band within the density gradient. Bands containing OMVs were collected, washed twice using sterile PBS at 100,000 × *g* for 2 h at 4°C and they were ultimately resuspended in 500 μL PBS. OMVs were divided into 0.5 mL aliquots and stored at −20°C until usage. These purified OMVs were used in all the experiments.

### Observation of OMVs Using Electronic Microscopy

Twenty-five microliters from the OMVs suspension (25 μg of protein) from both strains were placed onto copper grids coated with formvar and they were subsequently dried using filter paper. Then, 1% phosphotungstic acid was added onto the samples. The grids were allowed to dry for 10 h and they were observed with the aid of a transmission electron microscope (JEOL model JEM 10-10). In order to observe OMVs released from whole bacteria, cells from the *B. melitensis* strain 16M and *B. melitensis* VTRM1 were grown on TSA plates for 36 h at 37°C. Subsequently, molten soft agar was poured onto the plates to cover the growth. Once the agar had solidified, small agar cubes (2 mm) were cut. Blocks were fixed using 2.5% glutaraldehyde in PBS, rinsed with Sorensen’s PBS, dehydrated with ethanol, and prepared for transmission electron microscopy (TEM). Thin-section preparations were stained with OsO4, and they were observed using the JEOL model JEM 10–10, transmission electron microscope. Images were obtained using the aforementioned transmission electron microscope at ENCB’s (IPN, Mexico City, Mexico) Microcopy Facility.

### Denaturing Gel Electrophoresis (SDS-PAGE)

The SDS-PAGE protocol was performed using 15% acrylamide gel slabs, in accordance with Laemmli’s method (Laemmli, 1970). The gel slabs were stained with Coomassie blue and Silver Stain Plus Kit (Biorad). The apparent molecular masses of proteins contained in purified OMVs were determined by comparing their electrophoretic mobility against that of wide range molecular mass markers, using the SigmaGel^®^ V1.0 computer program.

### Liquid Chromatography Coupled to Mass Spectrometry in Tandem (LC-MS/MS)

After separation of proteins contained in OMVs through SDS-PAGE, the acrylamide gel was stained with Coomassie blue. The gel was then cut into four sections. Each gel section was analyzed as follows: Adjustment of the instrument parameters was performed with a Calmix solution containing *N*-butylamine, caffeine, Met-Arg-Phe-Ala (MRFA) and Ultramark 1621 (Pierce LTQ Velos ESI Positive Ion Calibration Solution). These reagents are used to calibrate the LTQ Veils module with ion trap and the Orbitrap module with FT (Fourier Transform) mass detector in the ESI positive ionization mode. The N-butylamine is used to extend the mass calibration at lower m/z (73.14 Da) values. This type of calibration allows for the determination of molecular masses with accuracy variations no higher than 5 ppm. Each gel section was reduced with dithiothreitol (Sigma-Aldrich; St Louis, MO, United States), which was alkylated with iodoacetamide (Sigma-Aldrich) and digested “in gel” with Trypsin (Promega Sequencing Grade Modified Trypsin; Madison, WI, United States). The digestion reaction was performed in a solution containing 50 mM ammonium bicarbonate (pH 8.2) and incubated for 18 h at 37°C. Peptides produced by enzymatic cleavage were desalinated using Zip Tip C18 (Millipore; Billerica, MA, United States) and applied into an LC-MS (Liquid Chromatography-Mass Spectrometry) system composed of an ACCELA pump (Thermo-Fisher, San Jose, CA, United States) coupled to an LTQ-Orbitrap Velos mass spectrometer (Thermo-Fisher Co., San Jose, CA, United States) with a nano-electrospray (ESI) ionization source. For the nano-flow liquid chromatography in line, a gradient system containing 5–80% solvent B (acetonitrile with 0.1% formic acid) was used with a set run-time of 120 min using an RP-C18 capillary column (0.75 μm internal diameter and 20 cm long). The system flow was set to 300 nL/min. The total ion scan (Full Scan) was performed on the Orbitrap analyzer with a resolution mass of 60,000. Peptide fragmentation was performed using the CID (Collision-Induced Dissociation) and the ion trap (IT) methods. These fragmentation methods are used because they provide more information about the peptide. All spectra were acquired using the positive detection mode. Execution and capture of fragmentation data were performed depending on the total ion scan according to pre-determined charges (ions with z2^+^, z^3+^, and z^4+^) charges were fragmented with an isolation width of 2.0 (m/z), normalized collision energy of 35 arbitrary units, Q activation of 0.250, activation time of 10 ms and maximum injection time of 10 ms per micro-scan. During automatic data capture, the dynamic ion exclusion was used as following: (i) 500 ion exclusion list, (ii) 30 s pre-exclusion time and (iii) 90 s exclusion time. Protein identification was performed by inputting spectrometric raw-data into the Proteome Discoverer v1.4 program. (Thermo-Fisher Co., San Jose, CA, United States) by means of the Sequest HT search engine. As for protein identity, the *B. melitensis* 16M genome was downloaded from the Uniprot database. An FDR-False Discovery Rate (Minimum) of 0.01 and FDR 0.05 (Maximum) was used in addition to the inverted database (Decoy database) as a tool for the “Percolator” validation program. The maximum tolerance of molecular mass difference for the precursor ion when compared to the theoretical versus experimental values (precursor mass tolerance) was 20 ppm and the tolerance for fragments obtained by dissociation of the precursor ion (fragment mass tolerance) was 0.6 Da. Constant modifications [carbamidomethylation of cysteines (C) and variables such as oxidation of methionines (M) and deamination of asparagine (N) and glutamine (Q)] were established for the automatic search. The samples were processed in duplicate.

### *In silico* Analysis of the Peptides Obtained From OMVs

Hits obtained from proteomic identification were analyzed with the aid of the by BlastP database, using the *B. melitensis* 16M genome (obtained from NCBI^1^). OrthoVenn2 analysis for enrichment of gene ontology terms was also used.^2^ Each protein along with its putative function was looked for in the PSORTb v3.0. ExPASy Bioinformatics Resource Portal^3^ and ProtCompB^4^ were used to determine the subcellular location of each protein. The protein Homology/analogy Recognition Engine V 2.0 (PHYRE2) database was used to find homologous proteins.^5^ The pathway enrichment was made using the KEEG database and the GhostKOALA tool.^6^

### Treatment of OMVs With Enzymes and Detergents

Outer membrane vesicles protect proteins that could be delivered to the host cells via fusion of the vesicles with the host cell or through endocytosis (McCaig et al., 2013). The purpose of this experiment was to evaluate resistance of OMVs against the effect of detergents and proteinase K. Purified OMVs (100 μg) from the *B. melitensis* 16M and VTRM1 strains were treated separately with 10 μL of proteinase K (Thermo Fisher Scientific) (10 mg/mL), SDS (0.02%), proteinase K 10 μL (10 mg/mL) plus SDS (0.5%) (Sigma-Aldrich, Inc), and sodium deoxycholate (0.5%) (Sigma-Aldrich, Inc). OMVs subjected to the different treatments were incubated at 37°C for 2 and 24 h. After incubation, phenylmethylsulfonyl fluoride (PMSF; Merck Millipore) (0.1 mM) was added to inhibit proteinase K, then all the samples were processed for SDS-PAGE analysis and subsequently stained with Silver Stain Plus Kit (Biorad) following the manufacturer’s recommendations.

### Isolation of Peripheral Blood Mononuclear Cells

Peripheral blood mononuclear cells (PBMCs) were separated from buffy coats provided by healthy donors. In short, blood units were diluted with sterile PBS and layered into a centrifuge tube with an equal volume of Ficoll-Paque Premium medium (GE Healthcare). Blood was centrifuged at 2, 000 × *g* for 25 min at room temperature, followed by two consecutive whases. The cells were then counted using a Neubauer chamber and adding Trypan blue. The cell suspension was adjusted a 1 × 10^6/^mL concentration in RPMI (Gibco^®^) supplemented with 10% fetal calf serum (FCS), 50 μg/mL gentamicin (Gibco^®^) and 2.5 μL/mL Fungizone (Gibco^®^). PBMCs platting was adjusted to 1 × 10^6/^mL/well, using a 24-well plate, and incubated under a CO_2_ atmosphere. After that, PBMCs were treated with different concentration of OMVs. For each experiment, PBMCs were obtained fresh and they were incubated overnight under same conditions. Healthy donors signed a written consent to participate in this study.

### Assessment of Apoptosis, DNA Damage and Cell Proliferation in PBMCs Treated With OMVs

The apoptosis, DNA damage and cell proliferation kit was used as per manufacturer’s instructions (BD Pharmingen^TM^). PBMCs were stimulated with either a 1, 10, and 25 μg/mL suspension of OMVs or 10 μL a of 1 mM Carbonyl cyanide 3-chlorophenylhydrazone solution (CCCP; Abcam) (de Graaf et al., 2004), the latter was used as a positive control for apoptosis. Furthermore, a 10 μg/mL suspension prepared using *Escherichia coli*-LPS (Sigma-Aldrich, Inc.) was used as a positive control for cell proliferation for a period of 24 h. Subsequently, bromodeoxyuridine (BrdU) was added to the cell suspension, followed by incubation for 1 h at a final 10 μM concentration. The cells were collected, washed using FACS buffer (PBS, 1% FCS, sodium azide 0.01%), and stained with monoclonal antibodies (mAbs) according to the manufacturer’s instructions: anti-human H2AX-Alexa Flour 647, and anti-cleaved PARP-PE. PARP (Poly [ADP-Ribose] Polymerase) is a nuclear chromatin-associated enzyme that is involved in DNA repair. During apoptosis, Caspase-3 cleaves PARP, resulting in its inactivation and a general inability of cells to repair DNA damage. For this reason, the 89-kDa-cleaved PARP fragment serves as a marker for cellular apoptosis. A total number of 50,000 events were acquired using the LSRFortessa cytometer (BD Biosciences), and the resulting data was analyzed with the aid of the FlowJo^®^ software v.10 (FlowJo, LLC, Ashland, OR, United States).

### Evaluation of Surface Markers of PBMCs Stimulated With OMVs

Peripheral blood mononuclear cells were stimulated with either a 1, 10, or 25 μg/mL suspension of OMVs from both strains or a 5 μg phytohemagglutinin (PHA) solution for 12, 24, and 48 h. Plates were incubated at 37°C under 5% CO_2_. Monoclonal antibodies (mAbs) coupled to the following fluorochromes were used: anti-human CD91-eFluor 660 (eBioscience), anti-human CD3-APC (BD Pharmingen^TM^), anti-human CD19-PE-Cy7 (BD Biosciences), anti-human PD-L1-PE (BioLegend), anti-human PD-1-FITC (BioLegend), anti-human CD86-PE (BioLegend), anti-human CD69-TRI-COLOR (Molecular Probes). After stimulation, 100 μL of supernatant were collected from wells and stored at −70°C until usage. Then, PBMCs were collected and washed with FACS buffer, followed by incubation with diluted mAbs in FACS buffer for 1 h at 4°C (protecting the reaction from light). Finally, cells were washed with FACS buffer, fixed with PBS-PFA (Paraformaldehyde) 1%, and resuspended in 400 μL of FACS buffer. Samples were analyzed in LSRFortessa^TM^ cytometer (BD Biosciences), providing a total number of 30,000 events, and the resulting data was analyzed with the aid of the FlowJo^®^ software.

### Immunomodulatory Effects of OMVs

In order to assess the potential regulatory effects of OMVs from *B. melitensis* on the expression of activation and inhibition surface markers, PBMCs were pre-incubated together with either OMVs or PHA. Briefly, 1 × 10^6^ cells^/^mL/well were incubated together with either 25 μg of OMVs from both strains or 5 μg of PHA with the supplemented medium at 37°C under 5% CO_2_ for a period of during 12 h. Then, the medium was replaced with freshly prepared supplemented medium. The cells that had been pre-incubated with OMVs were re-stimulated with 5 μg of PHA to evaluate the potential inhibitory effects of OMVs on the expression of surface markers. To determine possible regulatory effects of OMVs on the expression of the surface markers, cells pre-incubated with PHA were re-stimulated with 25 μg of OMVs from both strains. The expression of the activation (CD69, CD86) and inhibition (PD-1, PD-L1) surface markers were measured by flow cytometry as mentioned above.

### Cytokine Quantification

Cytokine quantification of Th1/Th2/Th17 cytokines; IL-2, IL-4, IL-6, IL-10, TNF, IFNγ, and IL-17A was performed with the aid of the CBA Human Th1/Th2/Th17 cytokine kit (BD Biosciences, San Diego, CA United States) and using the supernatants of cells that had been stimulated with either OMVs or PHA for 24 h, following the manufacturer’s instructions. Samples were read by using the LSRFortessa^TM^ cytometer (BD Biosciences) with CBA template, a total of 2000 events were recorded, and the resulting data was analyzed with the FCAP Array^TM^ software v3.0 (BD Biosciences).

### Effect of OMVs on PBMCs Cytoskeleton

Peripheral blood mononuclear cells were adjusted to 1 × 10^6^ and they were plated on sterile cover slips, followed by stimulation with 25 μg/mL of either OMVs or PHA for 2 and 6 h. Then, the cells were fixed using increasing concentrations of PFA (0.5, 1, and 2%) in PBS for 30 min. PBMCs were fixed onto cover slips by duplicated and permeabilized with Triton X-100 0.5% in PBS. Subsequently, one set of cells were treated with PBS/BSA 1% plus human serum 1% during 1 h at room temperature and it was later washed with PBS/BSA 0.2%. After that, 50 μL of mAb anti-tubulin (Biolegend) diluted 1:100 in PBS/BSA 1%, were added to the cells, to be incubated overnight at 4°C in a humid chamber. Cover slips were washed three times and incubated with mAb goat anti-mouse IgG Alexa fluor-488 (Invitrogen^TM^, Thermo Fisher Scientific Inc.) during 1 h at room temperature, under darkness. On the other hand, the other remained set of cover slips of fixed cells were incubated with rhodamine-phalloidin (Molecular Probes^TM^) diluted 1:100 in PBS during 30 min under darkness. Both sets of cover slips were washed and stained with DAPI 1 mg/mL diluted 1:10 in PBS for 1 min. Cover slips were washed and mounted using DABCO solution. Cells were analyzed with the aid of a confocal microscopy Leica TCS SP8 AOBS (Acousto-Optical Beam Splitter) DMI6000 (Leica Microsystems, Germany). A minimum of three fields were captured for analysis and nuclei were measured with ImageJ software.

### Ethics Statement

The present work was approved by the ethic committee of Escuela Nacional de Ciencias Biológicas, Instituto Politécnico Nacional with the approval number 4265. Written informed consent was signed by healthy donors.

### Statistics

Two-way ANOVA analysis with Bonferroni post-tests and One-way ANOVA analysis with Tukey/Dunnett post-test were used. GraphPad Prism V5.01 was used for statistical calculations.

## Results

### OMVs’ Purification and Treatment of OMVs With Proteinase K, Deoxycholate and SDS

**FIGURE 1:**
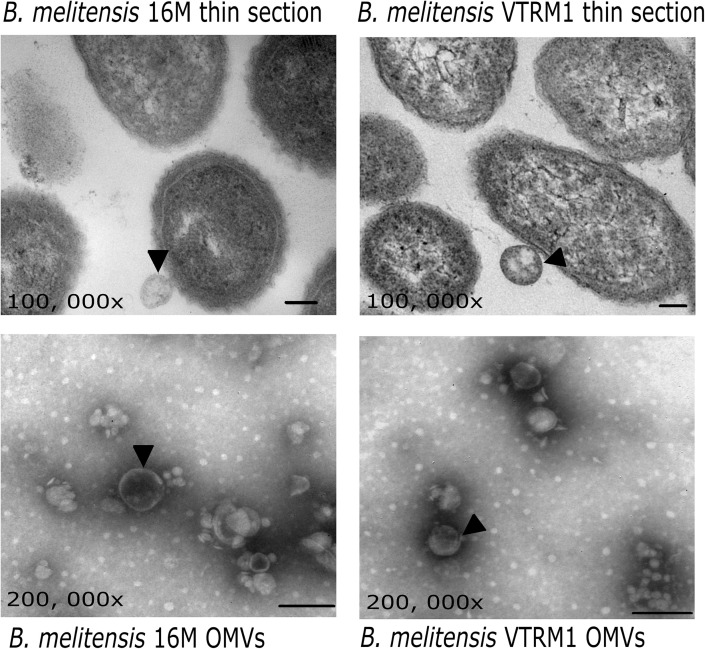
Electron microscopy of OMVs from *B. melitensis*. Micrographs show OMVs being released from *B. melitensis* 16M and *B. melitensis* VTRM1 cells (black arrowheads). OMVs stained with phosphotungstic acid evidence vesicles with a double membrane (black arrowheads). Bar = 100 nm.

**FIGURE 2:**
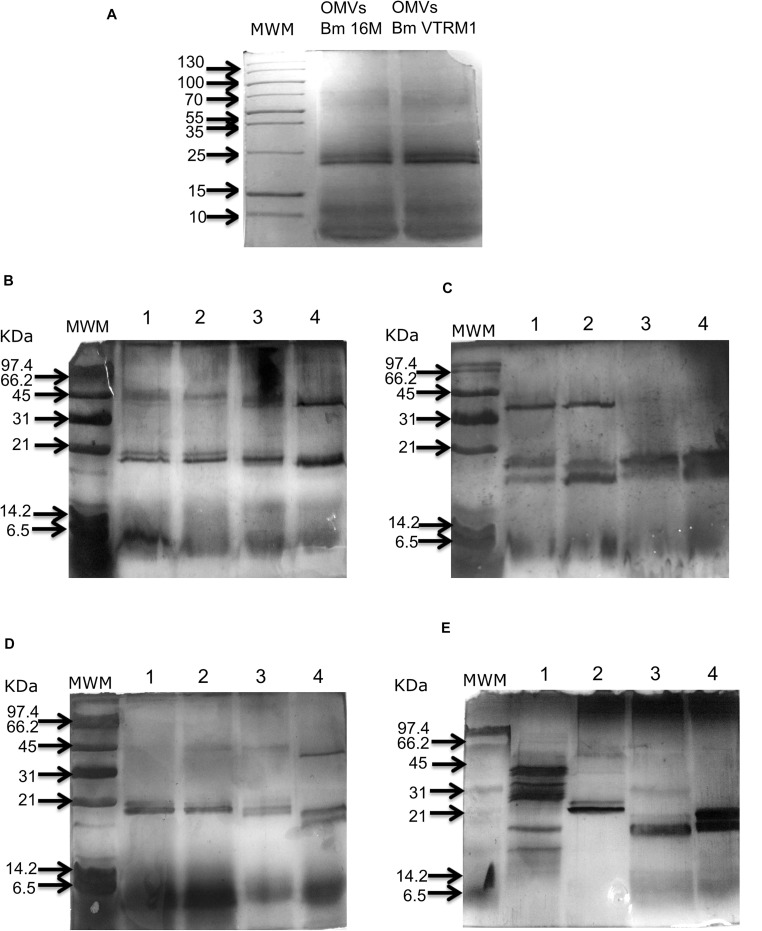
Protein profile obtained from *B. melitensis’* OMVs treated with detergents and proteinase K. **(A)** The protein profile of OMVs from *B. melitensis* 16M and VTRM1 strains exhibited similar profiles with two major bands of 23 and 26 kDa. **(B)** OMVs from *B. melitensis* 16M and **(C)** OMVs from *B. melitensis* VTRM1 were incubated at 37°C under different conditions for 2 h. **(D)** OMVs from *B. melitensis* 16M and **(E)** OMVs from *B. melitensis* VTRM1 were incubated at 37°C under different conditions for 24 h. The OMVs were treated with 10 μL proteinase K 10 mg/mL (lane 1), SDS 0.02% (lane 2), proteinase K together with SDS (lane 3), and deoxycholate 0.5% (lane 4). Denaturing electrophoresis was performed in 15% polyacrylamide slabs and stained with silver stain.

Outer membrane vesicles released from the whole cell of *B. melitensis* 16M and VTRM1 and purified vesicles were observed as showed in the micrographs (Figure 1). Protein profiles of OMVs purified from *B. melitensis* 16M and VTRM1 displayed bands ranging from 10 to 88 kDa, along with two notably major bands of 20 and 23 kDa (Figure 2A). OMVs from *B. melitensis* 16M treated with SDS, sodium deoxycholate and a mixture of proteinase K and SDS did not show changes in protein profile. Only a prominent band of 45 kDa was consistently observed in OMVs after both treatments (Figures 2B,D). A 45 kDa band was also observed in OMVs from *B. melitensis* VTRM1, treated with SDS and proteinase K (Figures 2C,E). The protein profile of OMVs of *B. melitensis* VTRM1, treated with proteinase K and SDS for 2 h, yielded two 16–18 kDa bands (Figure 2C). OMVs belonging to *B. melitensis* 16M, treated with deoxycholate, and a combination of proteinase K and SDS for 24 h showed a decreased in intensity of the bands corresponding to 18–20 kDa (Figure 2D). OMVs from the VTRM1 strain, treated with proteinase K for 24 h, displayed bands ranging from 14 to 65 kDa, while the resulting bands obtained from treatment with proteinase K together with SDS, showed several low-molecular-weight bands which are indicate the presence of partially degraded proteins. On the other hand, OMVs from the VTRM1 strain, treated with SDS or deoxycholate for 24 h, also affected proteins. However, two major bands in the vicinity of 18 and 20 kDa were still evident after SDS treatment (Figure 2E). In general, OMVs from *B. melitensis* 16M were more resistant to proteinase K as well as detergent treatment, whereas OMVs from *B. melitensis* VTRM1 were more sensitive.

### *In silico* Analysis

**FIGURE 3:**
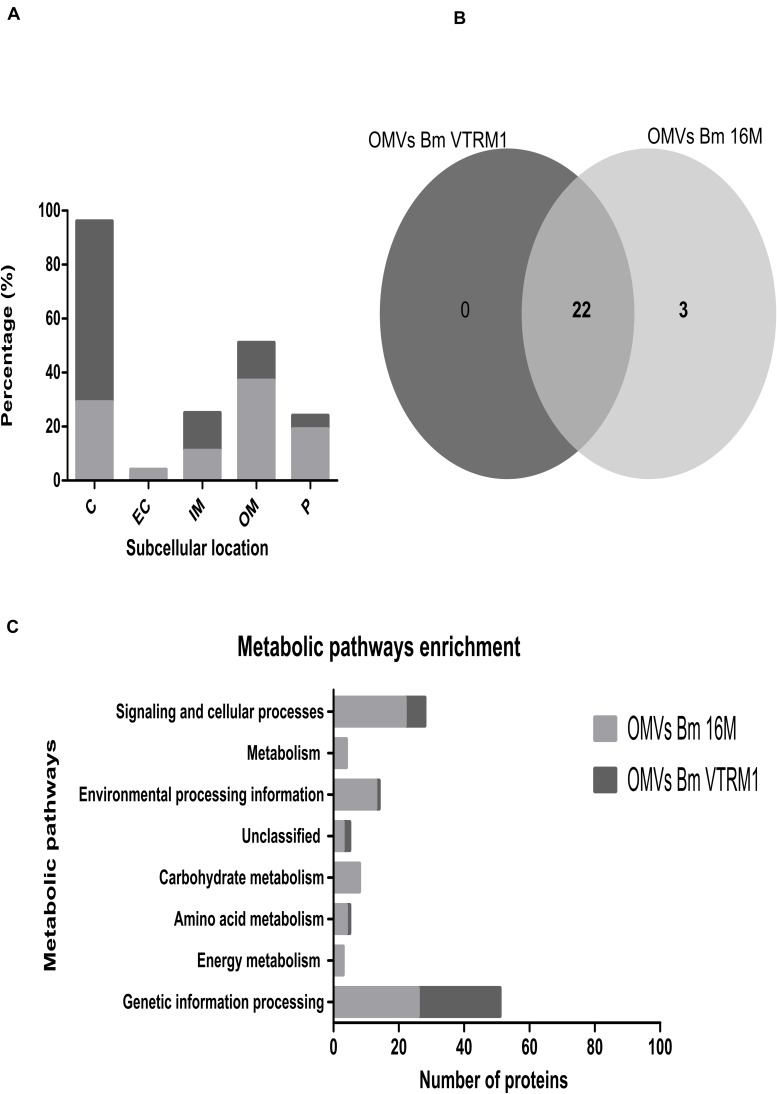
*In silico* analysis of peptides obtained from *B. melitensis*’ OMVS identified by LC-MS/MS. **(A)** After identification of peptides found in OMVs from *B. melitensis*, the peptide sequences were analyzed for protein location. Location of proteins of OMVs from *B. melitensis* 16M is highlighted in dark gray, while OMVs from *B. melitensis* VTRM1 in light gray. Periplasmic (P), cytoplasmic (C) outer membrane (OM) and inner membrane proteins (IM); **(B)** Orthologous proteins found in OMVs from the smooth and the rough strains were analyzed; the total proteins were grouped into 25 orthologous clusters for OMVs from the16M strain and 22 orthologous clusters for proteins found in the OMVs from the VTRM1 strain. A total of 22 clusters were shared by OMVs from both strains. **(C)** A metabolic pathway enrichment analysis showed a major enrichment pathway for proteins contained in OMVs.

The resulting peptide sequences obtained from the mass spectrometry analysis were used to scan through the databases. This analysis revealed 51 hits (proteins) for *B. melitensis* VTRM1 and 254 hits for *B. melitensis* 16M. A scan result was only accepted if the score or the coverage was higher than 20 and at least two tryptic peptides as well as their fragment ions matched the protein (Avila-Calderón et al., 2012, 2018). These hits were analyzed with the BlastP tool using the genomic sequence of *B. melitensis* 16M obtained from the NCBI. The proteins unambiguously identified were 131 for the *B. melitensis* 16M and 43 for the *B. melitensis* VTRM1 (Supplementary Table S1). The proteins contained in OMVs from both *B. melitensis* 16M and VTRM1 were identified according to aminoacid length, molecular weight (Mw), isoelectric point (pI), locus tag, subcellular location and COG (cluster of orthologous groups) functional classification (Supplementary Table S1). Supplementary Table S2, shows the proteins identified in two prominent bands obtained from the electrophoretic profile of OMVs from both strains, corresponding to 20 and 23 kDa. Any uncharacterized or hypothetical proteins lacking COG classification, were annotated along with the following information: string relation, protein domains (motifs) or homologous proteins found using the PHYRE2 analysis to characterize or assign a putative function. According to the subcellular location of proteins identified in OMVs, some notable differences regarding proportions were observed. Identified proteins in OMVs from the 16M strain were 29% cytoplasmic, 19% periplasmic, 37% outer membrane, 11% inner membrane, and 4% extracellular. In the case of OMVs from VTRM1, the identified proteins were 67% cytoplasmic, 5% periplasmic, 14% outer membrane, 14% inner membrane and no proteins from the extracellular location were found (Figure 3A). In order to compare the identified proteins found in OMVs from the smooth and rough strains, an analysis of orthologous proteins was performed. Proteins found in OMVs from *B. melitensis*16M were grouped into 25 clusters (22 proteins), while proteins contained in OMVs from *B. melitensis* VTRM1 were classified into 23 clusters. OMVs from both strains shared a total of 22 clusters, and only 3 clusters were exclusive for OMVs from *B. melitensis*16M (Figure 3B). The proteins shared in OMVs from both species are: Lon protease, Omp25, Omp31, and Omp16 (Supplementary Table S1). The 22 orthologous clusters shared in OMVs from both strains were grouped based on their biological-process ontology into five groups considering translation as the most important ontology group (GO: 0006412). Based on the molecular function process, orthologous proteins were grouped into nine groups and the most important ontologies were nucleic acid-binding (GO: 0003676) and structural molecule activity (GO: 0005198) (Supplementary Figure S1). Moreover, an uncharacterized orthologous protein was identified by this research team, BMEI0542, which showed a high degree of homology compared to OprG from *Pseudomonas aeruginosa* (98.8% confidence by PHYRE2). The latter protein has been associated to iron-uptake and cytotoxicity on the human bronchial epithelial cell line (McPhee et al., 2009) (Supplementary Table S1). A total of 103 proteins were found in OMVs from *B. melitensis* 16M and 21 proteins in OMVs from VTRM1 which were not classified into the orthologous clusters (singletons). The enrichment pathway showed a high number of proteins in OMVs from *B. melitensis* 16M that are related to genetic-information processing, environmental-processing information, signaling, cellular processes and carbohydrate metabolism. In the case of proteins identified in OMVs from the VTRM1 strain, these were mainly related to genetic-information processing, signaling and cellular processes (Figure 3C).

### OMVs Produced by *B. melitensis* Show No Cytotoxic Effects on PBMCs

It has previously been demonstrated that purified, lipidated-Omp19, from *B. abortus* 2308 induces apoptosis in T lymphocytes (Velásquez et al., 2012). On the other hand, *B. abortus* LPS induces DNA damage through the production of reactive oxygen species in polymorphonuclear cells (Barquero-Calvo et al., 2015). LPS and Omp19 are present in OMVs produced by *B. melitensis*, thus, both the analysis of apoptosis induction and DNA damage was performed. To determine whether OMVs display a cytotoxic-effect upon host cells, DNA damage and apoptosis were analyzed using PBMCs stimulated with OMVs from both *B. melitensis* strains. DNA damage was analyzed through the expression of the phosphorylated histone H2AX, while the induction of apoptosis was evaluated by measuring expression of the cleaved PARP [Poly (ADP-ribose) polymerase-1]. OMVs from both smooth and rough *B. melitensis* strains did not induce the expression of either cleaved PARP (cPARP) or phosphorylated H2AX on gated cells from the lymphocyte and monocyte region. Although gated cells from the monocyte region showed slight increase in cPARP expression, it was not statistically significant (one-way ANOVA with Dunnett post-test, 95% confidence interval) compared with the unstimulated control cells (Supplementary Figures S2A,B). These results demonstrated that OMVs show no cytotoxic effect in PBMCs. Moreover, OMVs did not induce cell proliferation in gated cells from the monocyte and lymphocytic regions (Supplementary Figure S2C).

### Pre-treatment Using OMVs From *B. melitensis* Modulates the Subsequent Expression of the Activation/Inhibition Surface Markers

**FIGURE 4:**
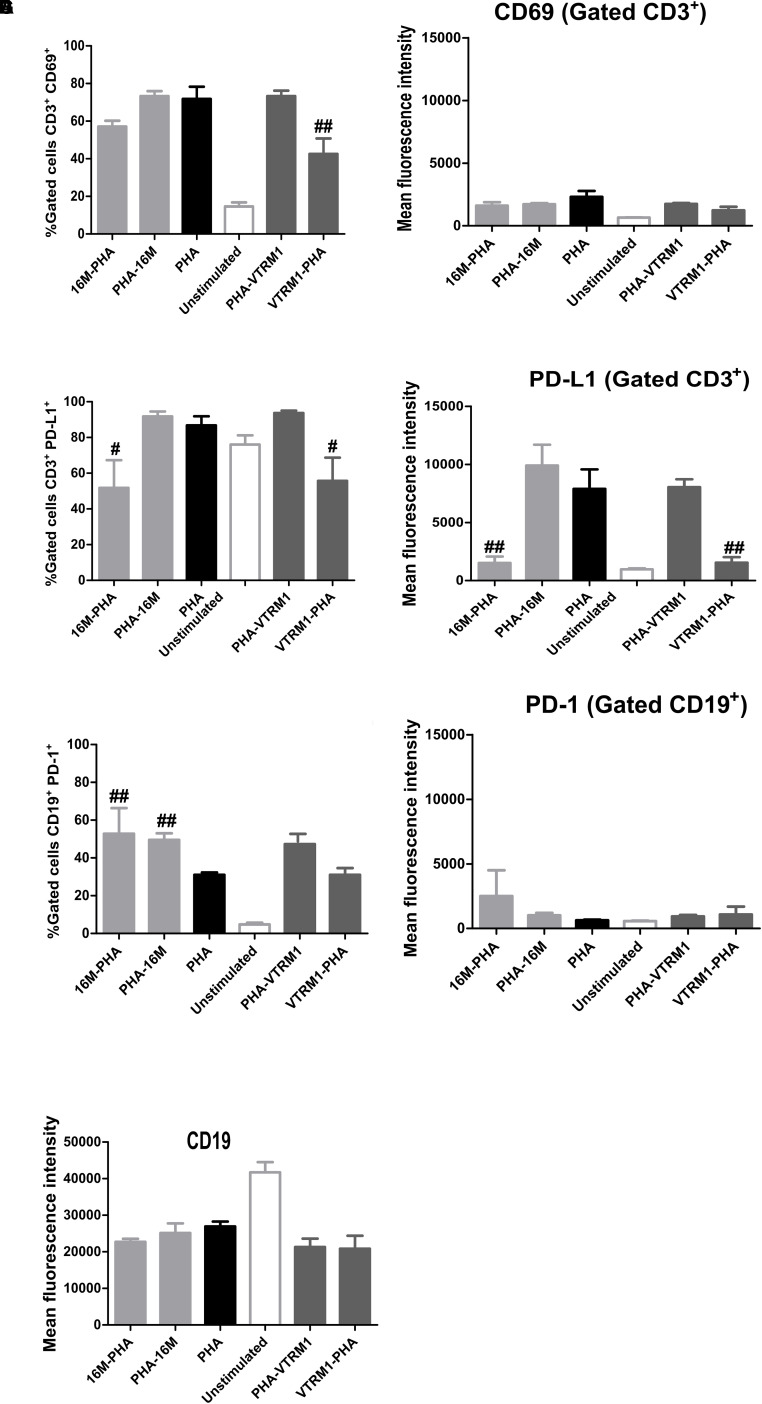
Immunomodulatory analysis of effects on the expression of activation/inhibition surface markers in PBMCs stimulated with OMVs from *B. melitensis*. PBMCs from healthy donor were stimulated using 25 μg/mL of OMVs from *B. melitensis* 16M and VTRM1 or PHA (5 μg/mL). Then, the preincubated cells with OMVs were re-stimulated with 5 μg of PHA in order to assess the inhibitory effect of OMVs on the expression of surface markers. Activation molecules were measured by flow cytometry using mAbs against CD69 and CD86 surface molecules and inhibition molecules were measured using mAbs against PD-1 and PD-L1. Expression was measured through mean fluorescence intensity (MFI) for each surface marker from the gated cells: monocytes (CD91^+^) and lymphocytes (CD3^+^, CD19^+^) and percentage of gated cells is also shown. Only the CD3^+^CD69^+^ gated cells pre-incubated with OMVs from VTRM1 strains showed a decrease in percentage **(A,B)**, whereas CD3^+^PD-L1^+^ cells incubated with both OMVs displayed a decrease in PD-L1 expression **(C,D)**. B cells were not affected by pre-incubation of OMVs, since no differences in the expression (MFI) of the surface markers were observed **(E–G)**. ^#^*P* < 0.05, ^##^*P* < 0.01, and ^###^ *P* < 0.001. ^#,##,###^Significant differences were observed in comparison with cells stimulated with PHA only.

Sonicated *B. melitensis* Rev1 induced lymphocyte activation through CD69 expression (Almajid, 2011). On the other hand, recombinant Omp25, identified in OMVs from *B. melitensis*, decreased IL-12 production through PD-1 signaling in monocyte/macrophages (Cui et al., 2017). Thus, it was expected that OMVs produced by *B. melitensis* induce the expression of surface activation or inhibition markers. Results showed no differences in the expression of either the activation (CD69 and CD86) or inhibition (PD-1 and PD-L1) markers on PBMCs stimulated with OMVs at the different time intervals tested (two-way ANOVA with Bonferroni post-test, 95% confidence interval) (Supplementary Figures S3, S4). The percentage of CD3^+^CD69^+^, CD19^+^CD69^+^, CD3^+^PD-1^+^, and CD19^+^PD-1^+^ cells showed an increase after 24 h and a decrease 48 h after treatment with different concentrations of OMVs; however these changes were not statistically significant. To determine how the manner in which OMVs from *B. melitensis* regulate the expression of activation and inhibition cell surface markers on PBMCs, the cells were pre-incubated with OMVs before a subsequent PHA-treatment, which showed that the number of T cells (CD3^+^) expressing CD69 decreased. It should be noted that significant differences was observed only when cells were treated with OMVs from the VTRM1 strain compared with cells stimulated with PHA in absence of OMVs (*p* < 0.005) (one-way ANOVA with Tukey post-test, 95% confidence interval). Additionally, the number of T cells expressing the PD-L1 marker decreased significantly when PBMCs were pre-incubated with OMVs from both strains, in comparison with PHA stimulated cells (*p* < 0.05) (Figures 4A–D). Pre-incubation with OMVs not only decreased the number of CD3^+^ PD-L1^+^ cells, but also the expression level of the PD-L1 molecule expressed by mean fluoresce intensity (MFI) in gated T-cells (*p* < 0.05) (Figures 4B,C). Conversely, incubation of PBMCs with OMVs from *B. melitensis* 16M prior to or after the stimulation with PHA, increased the number of B cells (CD19^+^) expressing PD-1, in comparison with the positive control (*p* < 0.005) (Figures 4E–G). Therefore, OMVs seem to exert an inhibitory effect on the expression of the surface markers in both T and B cells, which in turn modulates the expression of the CD69 and PD-1/PD-L1 molecules. OMVs decreased the number of CD3^+^ CD86^+^, CD3^+^ PD-1^+^, and CD91^+^ PD-1^+^ cells but not to a significant degree (Supplementary Figures S5, S6).

### OMVs Produced by *B. melitensis* Inhibit the Th17 Cytokine but Increase Production of TNFα/IL-6

**FIGURE 5:**
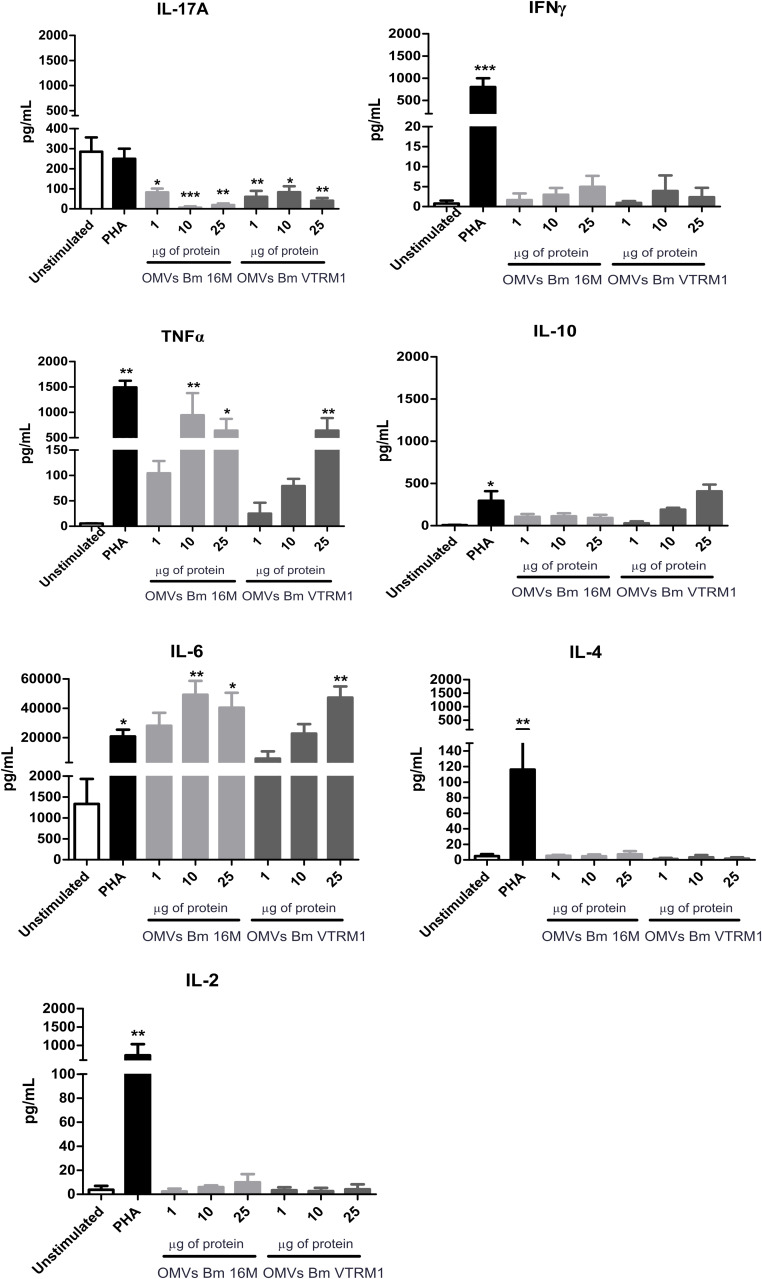
Cytokine quantification from PBMCs stimulated with *B. melitensis* OMVs. Cytokines were determined using the CBA cytometric assay at different time intervals. PHA was used as positive control and Th1/Th2/Th17 cytokines levels were measured. *P < 0.05, **P < 0.01, and ***P < 0.001. *Significant differences were observed in comparison with the unstimulated cells.

To determine whether the vesicles drive the immune response through a Th1, Th2 or Th17 profile, the production of each specific cytokine was measured. Figure 5 shows that OMVs produced by both *B. melitensis* strains did not induce the production of either the Th2- (IL-4 and IL-10) or Th1-type cytokines (IL-2, IFNγ) (one-way ANOVA with Tukey post-test, 95% confidence interval). IL-17A decreased in the presence of different concentrations of OMVs, while production of TNFα and IL-6 increased using 10 and 25 μg/mL of OMVs from *B. melitensis* 16M (*p* < 0.005, *p* < 0.05 respectively), and 25 μg/mL of OMVs from the rough VTRM1 strain induced cytokine production (*p* < 0.005) (Figure 5).

### OMVs Induce Actin and Tubulin Depolymerization in PBMCs

**FIGURE 6:**
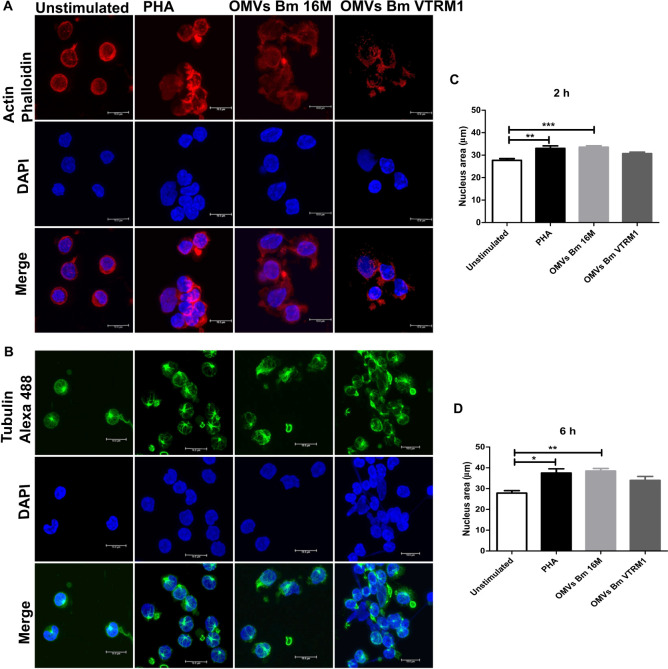
Cytoskeleton rearrangement and morphological chances in PBMCs induced by OMVs from *B. melitensis*. After 6 h post-stimulation with *B. melitensis* OMVs from the smooth and rough strains, morphologic changes in PBMCs were observed compared with unstimulated PBMCs. Confocal microscopy showed defective actin **(A)** and tubulin **(B)** polymerization in PBMCs stimulated with OMVs from both strains. Stimulation of PBMCs with OMVs from smooth and rough *B. melitensis* strains induce morphological changes in the nucleus at 2 h **(C)** and 6 h **(D)** of stimulation. Cells stimulated using OMVs from both *Brucella* strains displayed enlarged nucleus shaped. *P < 0.05, **P < 0.01, and ***P < 0.001. *Significant differences were observed in comparison with unstimulated cells. Bar = 10 μm.

It has previously been reported that *Brucella* is able to induce cytoskeleton rearrangements (Czibener et al., 2016). In order to evaluate the effect of OMVs release by *Brucella* on the cytoskeleton ultrastructure, PBMCs were treated with OMVs and noticeable morphological changes were observed by confocal microscopy. After incubation with OMVs for 2 h, PBMCs showed small actin foci within the cells and the nucleus showed a different affinity to the DAPI stain (Figures 6A,B). After 6 h stimulation with OMVs, the PBMCs showed evident actin and tubulin depolymerization, as well as morphological changes in the nucleus. The observed cells displayed an enlarged nucleus and different affinity for the DAPI stain. Moreover, the microtubule-organizing center was not well-defined in some cells that had been stimulated with OMVs (Supplementary Figures S7A,B). Even though these changes were observed in cells treated with OMVs from both strains, the effect was more evident in cells treated with OMVs from the rough strain. The nucleus areas of the treated cells were measured and after incubation with OMVs for about 2 h, the nuclei exhibited a significant increase in area size, though only when using OMVs from the smooth strain (2 h, *p* < 0.001; 6 h, *p* < 0.005) (Figures 6C,D) (one-way ANOVA with Tukey post-test, 95% confidence interval).

## Discussion

Outer membrane vesicles from *B. melitensis* were first observed by Gamazo and Moriyon (1987) using samples from the smooth, *B. melitensis* 16M and the rough, *B. melitensis* 115 strains, both of which had been cultured in tryptic soy broth. The protein profile of OMVs from *B. melitensis* 16M and VTRM1 obtained in this study showed two major bands corresponding to 25 and 30 kDa along with other less-intense bands corresponding to 18, 22, and 84 kDa. These results were similar to those reported for protein profiles of OMVs from *B. melitensis* 16M and *B. melitensis* 115 rough strain cultured in liquid tryptic soy broth (Gamazo and Moriyón, 1987). However, different protein profiles have been observed in *B. melitensis* strains cultured under different conditions. For instance, Gamazo et al. (1989) analyzed proteins contained in OMVs from *B. melitensis* strains, isolated from humans and animals and cultured on blood agar plates. Protein profiles of these OMVs were obtained by SDS-PAGE electrophoresis. They observed proteins grouped as following: 67-30 kDa (designated as group A), 21.5 kDa (group B), 19.5-18.2 kDa (group C), and 15-13.5 kDa (group D). Jain-Gupta et al. (2012) observed a great number of bands ranging from about ∼15 up to ∼70 kDa in the electrophoretic profile of OMVs from *B. melitensis* 16M cultured on tryptic soy plates. In 2019, a recent study by Bagheri-Nejad obtained OMVs from a clinical human isolate, the pathogen was identified as *B. melitensis* biovar 1, which was cultured on *Brucella*-agar medium. They observed in the protein profile bands corresponding to sizes smaller than 20 kDa and a bigger band than 25 kDa in OMVs (Bagheri-Nejad et al., 2019).

The electrophoretic protein profiles of OMVs from *B. melitensis* 16M and VTRM1, obtained in the present work, were similar to those reported in Avila-Calderón et al. (2012). Nonetheless, the methodology to obtain the vesicles were different in both works: a density-gradient centrifugation was used for purification of OMVs in this work, whereas Avila-Calderón et al. (2012) only used ultracentrifugation steps without a density gradient. It should be mentioned that, this difference in methodology did not impact the protein profile obtained from OMVs. It is known that culture conditions *in vitro* define the protein content of OMVs (Sidhu et al., 2008; Choi et al., 2014). In this work as well as the one published by Avila-Calderón et al. (2012), culture conditions for *Brucella* strains were the same.

In last few decades, proteomics has become a powerful tool in the identification of proteins contained within OMVs. A total of 131 proteins were identified in OMVs purified from the *B. melitensis* 16M smooth strain, and 43 in OMVs from the *B. melitensis* VTRM1 rough strain, employing an LTQ orbitrap-Velos mass spectrometer. Avila-Calderón et al. (2012) identified 49 proteins in OMVs from *B. melitensis* 16M using the Finnigan LCQ ion trap mass spectrometer, though the identification of proteins contained in OMVs from *B. melitensis* VTRM1 was not performed. Differences found in the number of proteins detected in OMVs from *B. melitensis* 16M could be attributed to the use of different equipment. In 2019, a recent study by Araiza-Villanueva et al. (2019) reported 228 proteins identified in OMVs from *B. abortus* 2308 and 171 in membrane vesicles from the *B. abortus* rough strain RB51.

In all three studies: Omp25, Omp31, SodC, Omp19, and Omp16 among others were found in OMVs from *Brucella* among others. Boigegrain et al. (2003) had previously identified Omp25 and Omp31 in OMVs from *B. suis* 1330. In 2019, a recent study by Araiza-Villanueva et al. (2019) identified 171 proteins found in vesicles from the *B. abortus* RB51 rough-vaccine strain, while vesicles from the *B. abortus* 2308 smooth strain, yielded a total of 228 proteins. In the present work, a less number of proteins were identified in OMVs from *B. melitensis* VTRM1 rough mutant, in comparison to proteins found in OMVs from the *B. melitensis* 16M smooth reference strain. *B. abortus* RB51 is a rough strain used as a live attenuated vaccine to prevent brucellosis in cattle. *B. abortus* RB51 is a rough strain derived from *B. melitensis* 2308, lacking glucosyl transferase activity (responsible for O-side chain synthesis), obtained by disruption of the *wbo*A gene (Schurig et al., 1991; Avila-Calderón et al., 2013). *B. melitensis* VTRM1 is a stable rough-mutant derived from the *B. melitensis* 16M strain, obtained by allelic exchange of the *rfb*U gene (encoding for a mannosyltransferase), which is known to induce protection against virulent *Brucella* strains (Winter et al., 1996). Some findings in bacterial LPS mutants have contributed to a better understanding in the role played by LPS on protein selection for the assembly of OMVs. For example, OMVs from *Klebsiella pneumoniae wbbO* mutant (unable to synthesize the O-side chain), displayed a different protein composition compared with OMVs from the wild-type strain which contain complete LPS (Cahill et al., 2015). OMVs purified from the *K. pneumoniae* wild-type strain contained proteins involved in cell wall, membrane, as well as envelope biogenesis, while OMVs from the *K. pneumoniae wbbO* mutant carried proteins associated with post-translational modification, protein turnover, and chaperones (Cahill et al., 2015). OMVs from the *B. melitensis* VTRM1 rough strain a exhibited higher concentration of proteins involved in genetic-information processing, signaling and cellular processes. In contrast, OMVs from the *B. melitensis* 16M smooth strain contained proteins engaged in genetic-information processing, environmental-processing information, signaling, and cellular processes. Therefore, the O-side chain belonging to LPS could define the type and number of proteins packed in *Brucella* vesicles.

Outer membrane vesicles from a *P. aeruginosa* OSA-mutant contained periplasmic proteins and little OMPs, whereas OMVs from a *P. aeruginosa* CPA-mutant contained a higher number of OMPs and little periplasmic proteins (Murphy et al., 2014). OSA, is a negatively charged O-specific antigen and it is highly immunogenic, while CPA is a short *O*-antigen referred to as common polysaccharide antigen in *P. aeruginosa* (Lam et al., 2011). OMVs from *B. melitensis* 16M displayed a high number of OMPs, cytoplasmic and periplasmic proteins. Contrastingly, OMVs from *B. melitensis* VTRM1 contained mainly cytoplasmic proteins; OMPs and proteins from the inner membrane at a similar ratio. The negative charge of *Brucella*-LPS is mainly located at the core, not at the O-side chain. The *wadC* gene encodes for a glycosyltransferase involved in the synthesis of the core oligosaccharide branch, which is not linked to the *O*-antigen, and it has a positive charge that balances internal, negative LPS charges (Soler-Lloréns et al., 2014; Fontana et al., 2016). Probably an O-side chain lacking in *B. melitensis* VTRM1 (rough mutant), may be responsible for a charge imbalances on the surface of *Brucella*, affecting protein composition, the number of proteins and the proportion in the location of proteins within OMVs derived from this rough strain.

Lipopolysaccharides structure integrity may not only be affected by the mechanisms for protein sorting but also by the resistance of OMVs to detergents and enzymes. Incubation of vesicles along with proteinase K results in degradation of surface proteins, while SDS impairs the integrity of OMVs, allowing for the access of the protease into the vesicle lumen (McCaig et al., 2013). The protein profile of OMVs from *B. melitensis* 16M displayed minimal changes after a 24 h treatment with either detergents or proteinase K and combination of both. While, OMVs from *B. melitensis* VTRM1, which lacks the O-side chain, were in fact affected and showed a different profile after treatment. OMVs from *B. melitensis* VTRM1, treated with proteinase K for 24 h, showed a higher number of protein bands, in comparison with non-treated OMVs for the same period of time. Similar results had previously been reported in OMVs from *Francisella novicida* treated with proteinase K. Results showed that surface proteins were degraded, while SDS impaired the integrity of the outer membrane, allowing for the access of proteinase K into the vesicle lumen, which led to changes of the protein profile obtained from the electrophoretic profile (McCaig et al., 2013). Quite possible, the protein profile observed in OMVs from the rough-mutant VTRM1 may correspond to proteins from the surface and lumen of OMVs which were extracted with detergents and then degraded by proteinase K. These results clearly demonstrated differences between OMVs from the smooth and rough *Brucella* strains, while OMVs from the rough strain were affected by detergents and proteinase K. The presence of complete LPS in OMVs derived from the smooth strain somehow protected the protein content. LPS plays a decisive role in determining the protein content of OMVs and it also protects bacteria from cationic antimicrobial peptides, reactive oxygen species and complement-mediated lysis (Lapaque et al., 2005). OMVs from smooth *Brucella* strains transport proteins (including virulence factors) which can be protected by complete LPS. Lamontagne et al. (2007) were able to identify the Omp31b and Omp25 in membrane fragment from *B. abortus* using mass spectrometry. Moreover, they showed that *B. abortus* altered the membrane-protein expression pattern after macrophage infection. For instance, the concentration of GroEL, SodC and membrane transport increased in OMVs from *B. melitensis* under oxidative stress, during macrophage infection (Lamontagne et al., 2009). Thus, OMVs may serve as a delivery vehicle for virulence factors into host cells.

Outer membrane vesicles from both *Brucella* strains studied in the present work, neither induced apoptosis mediated by cleaved PARP nor the expression of histone H2AX (DNA damage). Additionally, different concentrations of OMVs from both strains did not induce the expression of activation (CD69 and CD86) or inhibitory (PD-1, PD-L1) markers. However, pre-treatment of PBMCs with OMVs from *B. melitensis* 16M and VTRM1 decreased the number of CD3^+^CD69^+^ and CD3^+^PD-L1^+^, whereas only OMVs from *B. melitensis* 16M increased the number of CD19^+^PD-1^+^ cells. Although OMVs from *B. melitensis* 16M and VTRM1 decreased the numbers of CD3^+^CD69^+^, the effect was significant only with OMVs from *B. melitensis* VTRM1. It appears that OMVs from *B. melitensis* inhibit T-cell responses. Programmed death-1 (PD-1) and their corresponding ligand programmed death ligand-1 (PD-L1) are molecules belonging to the B7/CD28 superfamily and they have previously been described to play an inhibitory role on lymphoid and myeloid cells. PD-1/PD-L1 regulates T cell function by inhibition of the proliferation and cytokine regulation (Hofmeyer et al., 2011). However, PD-1/PD-L1 role appears to be a source of discussion, since some authors have reported an inhibitory role for these surface molecules, and others have reported a positive role in the immune response. For instance, after mice are infected with *Listeria monocytogenes*, PD-1 and PD-L1 were up-regulated in CD4^+^, CD8^+^, NK cells, and macrophages. Inactivation of PD-L1 by mAb, increased susceptibility to lethal bacterial doses, inhibited production of TNFα as well as nitric oxide in macrophages and production of IFNγ in NKC cells (Seo et al., 2008). Considering the case of infected cattle with *Mycoplasma bovis*, animals showed proliferation of CD4^+^PD-1^+^ and CD4^+^ PD-L1^+^ cells, decreasing IFNγ production, and the inactivation of the PD-1 and PD-L1, using mAbs restored IFNγ production in PBMs from infected animals (Goto et al., 2017). PD-1 expression has been related to exhausted CD8 + T cells in chronic brucellosis (Durward-Diioia et al., 2015). Recent studies, have demonstrated up-regulation of PD-1in THP-1 cells, induced by recombinant *B. suis* Omp25. The up-regulation of PD-1 and PD-L1 in THP-1 cells induced the expression of microRNAs (miRNAs) that inhibits IL-12 production. The inactivation of PD-1 decreases miRNAs expression. Although PD-1/PD-L1 have been related to miRNAs expression, only the PD-1 pathway modulated IL-12 cytokine levels (Cui et al., 2017). The present work revealed a decrease in the number of CD3^+^PD-L1^+^ in PBMCs pre-treated with OMVs from both *Brucella* strains studied, as well as inhibition in the expression (MFI) of the PD-L1 marker in gated T-cells. Furthermore, pre-treatment and re-stimulation with OMVs from *B. melitensis* 16M increased the number of CD19^+^PD-1^+^. Xu et al. (2013) described a costimulatory effect of PD-L1 in CD8^+^ T-cells against *L. monocytogenes* infection, and this effect may be attributed to interactions with an unknown receptor. Likewise, an increase of bacterial clearance was observed in the liver and spleen of mice infected with *L. monocytogenes*, treated with blocked PD-1 (Xu et al., 2013). Based on these results it was proposed that costimulatory and inhibitory roles of PD-L1 and PD-1 occurred simultaneously, while PD-L1 blockage reduced CD8^+^ against *L. monocytogenes* infection, PD-1 blockage improved elimination of bacteria without altering CD8^+^ T-cells. Considering the interactions found among PBMCs and OMVs *Brucella*, it is proposed that PD-L1 inhibition on CD3^+^ cells is related to T-cell malfunction or poor T-cells activity. This inhibitory effect was independent on the presence of the O-side chain, being that OMVs from the *B. melitensis* 16M smooth strain and VTRM1 rough mutant led to a decrease in the CD3^+^PD-L1^+^ percentage and MFI values. The inhibitory effect of OMVs from *B. melitensis* was observed directly on T-cell populations; that is, the pre-treatment of PBMCs with OMVs neither reduced nor increased the numbers of B cells expressing activation/inhibition surface markers through a second stimulus (PHA). Clearly, OMVs do not affect the expression (MFI) of surface activation/inhibition or CD19 markers (Supplementary Figure S3) on B cells.

Hence, after PBMCs were stimulated with PHA, either an increase or null-effect in the number of B cell populations like CD19^+^PD-1^+^ cells could be observed. Conversely, pre-treatment with OMVs from *Brucella* either inhibited or reduced the number of T-cells expressing the activation/inhibition surface markers after exposure to the second stimuli, supporting the idea that OMVs from *B. melitensis* of both strains exert an inhibitory effect on lymphocytic cells.

Stimulation of PBMCs using OMVs produced by both *B. melitensis* strains induced high concentrations of IL-6 and TNFα, but inhibited IL-17A production. IL-6 is a pleiotropic cytokine involved in inflammatory and anti-inflammatory responses. *B. abortus* 2308 cells and *B. abortus* Omp19 down-regulated the IFNγ-induced MHC-II expression via IL-6 secretion in THP-1 monocytes (Velásquez et al., 2016). TNFα is an important cytokine in Th1 immune responses to eliminate intracellular pathogens. Lou et al. (2018) showed TNFα down-regulation on the expression of miRNAs induced by cells of *B. suis* and *B. suis* Omp25 protein in porcine and murine macrophages. Other *Brucella* antigens such as the Lon protease, phosphoglyceromutase, and dihydrodipicolinate reductase induce TNFα production (Park et al., 2013; Li et al., 2018). IL-17A has been found at high concentrations in patients with acute brucellosis (Sofian et al., 2016). IL-17 has been described to be involved in osteoclastogenesis; T CD4+ cells were activated with supernatant from peritoneal macrophages infected with *B. abortus*, activated T cells induced osteoclastogenesis in bone marrow macrophages via IL-17 (Giambartolomei et al., 2012). OMVs from *B. melitensis* VTRM1 induced TNFα expression in murine BMDCs, but OMVs from *B. melitensis* 16M did not. Moreover, IL-6 and IL-17 expression was observed early (1 h) in cells stimulated with OMVs from *B. melitensis* VTRM1, whereas cells stimulated with OMVs from *B. melitensis* 16M expressed both cytokines after 12 h (Avila-Calderón et al., 2012). Th17 cells are induced by IL-6 and TGFβ, and Th17 differentiation requires signal transducer and activator of transcription STAT-3 activation and suppression of STAT1 (Kimura and Kishimoto, 2010). IL-6 signals through the Janus family tyrosine kinases (JAK1, JAK2) and gp130 and induce STAT1 and STAT3 activation (Naka et al., 2002). It is possible that, an IL-6 overproduction in PBMCs stimulated with OMVs from *B. melitensis* induced strong STAT1 activation, inhibiting Th17 and subsequently IL-17 production.

Results demonstrated actin and tubulin cytoskeleton rearrangements in PBMCs stimulated with OMVs from *B. melitensis* after stimulation period of 6 h. The cytoskeleton is involved in a variety of cellular processes such as intracellular trafficking and signaling, among others (Gundersen and Cook, 1999). It has previously been demonstrated that *Brucella* is able to induce cytoskeleton rearrangements; for instance, BigA, an adhesion protein containing the immunoglobulin-like domain found in *B. abortus*, induces actin-cytoskeleton rearrangement in Caco-2 and the dog cell line MDCK (Czibener et al., 2016). Alves-Silva et al. (2017) demonstrated that BtpA induced microtubule bundling in bone-marrow derived macrophages (BMDM) and increased *B. abortus* virulence. In addition, BMDM stimulated with BtpA and infected with *B. abortus* induced IL-12 and IL-1β production (Alves-Silva et al., 2017). Cytoskeleton rearrangement and TNFα production have been linked to cytokine production by way of common signal pathways such as ERK kinases, a component of the mitogen-activated protein kinase (MAPK) signaling pathway (Rosengart et al., 2002). Based on the results of this work, it can be proposed that TNFα and/or IL-6 overproduction may be involved in cytoskeleton rearrangements in PBMCs stimulated with OMVs from *B. melitensis*. Moreover, it is possible that uncharacterized antigens carried within OMVs from *Brucella* could induce the effect on the cytoskeleton. The orthologous hypothetical membrane-associated protein (BMEII0692) (Accession Q8YC40) was found in OMVs from *B. melitensis* 16M and VTRM1, and possesses an invasion associated locus B (IalB) protein motif (15–147 aa), and it is highly homologous to the IalB protein from *Bartonella henselae* (Supplementary Table S1). *B. henselae* IalB is located at the outer membrane and is required for erythrocyte invasion (Deng et al., 2016). *Brucella* hypothetical protein BMEII0692 or the BMEI0542 homologous to OprG from *P. aeruginosa* should be further analyzed for their role in *B. melitensis* infection.

## Data Availability Statement

The proteomic data analyzed in this study was obtained from Proteomic Facility at Instituto de Biotecnología Universidad Autónoma de México, the dataset is restricted by the third party. Requests to access these datasets should be directed to Unidad de Proteómica, upro@ibt.unam.mx. Flow cytometry data is available at the following links: http://flowrepository.org/experiments/3065, http://flowrepository.org/experiments/3067, http://flowrepository.org/experiments/3068, http://flowrepository.org/experiments/3066.

## Ethics Statement

The studies involving human participants were reviewed and approved by Comité de Ética de la Escuela Nacional de Ciencias Biológicas del Instituto Politécnico Nacional. The patients/participants provided their written informed consent to participate in this study.

## Author Contributions

EA-C, AC-R, AL-M, and LF-R conceived and designed the experiments. EA-C, AC-R, OM-C, JH-H, and ZG-L performed the experiments. EA-C, ER, MA-A, and BA-R analyzed the data. EA-C, MA-A, LF-R, SW, and AC-R wrote the manuscript. LF-R and MA-A gave practical suggestions to perform the experiments. All the authors contributed to the article and approved the submitted version.

## Conflict of Interest

The authors declare that the research was conducted in the absence of any commercial or financial relationships that could be construed as a potential conflict of interest.

## Footnotes

1 http://www.ncbi.nlm.nih.gov

2 https://orthovenn2.bioinfotoolkits.net/home

3 http://www.psort.org/psortb/index.html

4 http://linux1.softberry.com/berry.phtml?topic=pcompb&group=programs&subgroup=proloc

5 http://www.sbg.bio.ic.ac.uk/∼phyre2/html/page.cgi?id=index

6 https://www.kegg.jp/ghostkoala/

## Supplementary Material

Supplementary Figure 1 GO enrichment analysis of identified proteins in OMVs from *B. melitensis*. The orthologous proteins shared between both OMVs were analyzed for functional classification. Orthologous proteins were grouped based on their biological process ontology into five groups, being the most important ontology, translation (GO: 0006412). Based on molecular function process the orthologous proteins were grouped into nine groups and the most important ontologies were nucleic acid binding (GO: 0003676) and structural molecule activity (GO: 0005198).

Supplementary Figure 2 Apoptosis, DNA damage and cell proliferation of PBMCs induced by OMVs from *B. melitesis*. PBMCs from healthy donor were stimulated with different concentrations of OMVs from *B. melitensis* for 24 h. Apoptosis, DNA damaged and cells proliferation were evaluated by flow cytometry with mAbs anti-PARP, anti-H2AX and-BrdU. CCCP was used as control for apoptosis induction, while *E. coli* LPS 10 μg/mL was used as control for proliferation. ^∗^*P* < 0.05, ^∗∗^*P* < 0.01, and ^∗∗∗^*P* < 0.001. ^∗^Significant differences were observed in comparison with the unstimulated cells (two-way ANOVA with Bonferroni post-test).

Supplementary Figure 3 Analysis of expression of activation/inhibition surface markers in PBMCs stimulated with *B. melitensis* OMVs. PBMCs from healthy donors were stimulated with 1, 10, and 25 μg/mL of OMVs from *B. melitensis* 16M and VTRM1 strains during 12, 24, and 48 h. Activation markers were measured by flow cytometry using mAbs against surface molecules CD69 and CD86 and inhibition markers using mAbs against PD-1 and PD-L1. Expression was measured with the Mean fluorescence intensity (MFI) of each surface marker from the gated cells: monocytes (CD91^+^) and lymphocytes (CD3^+^, CD19^+^). PHA (5 μg/mL) was used as control for the expression of surface markers. ^∗^*P* < 0.05, ^∗∗^*P* < 0.01, and ^∗∗∗^*P* < 0.001. ^∗^The significant differences were observed against the unstimulated cells.

Supplementary Figure 4 Analysis of the surface expression of activation/inhibition markers in PBMCs stimulated with OMVs from *B. melitensis*. PBMCs were stimulated with either a 1, 10, or 25 μg/mL suspension of OMVs from both strains or a 5 μg phytohemagglutinin (PHA) solution for 12, 24, and 48 h. Activation was measured by flow cytometry using mAbs against surface molecules CD69 and CD86 and inhibition was measured using mAbs against PD-1 and PD-L1. The percentage of total gated cells monocytes (CD91^+^) and lymphocytes (CD3^+^, CD19^+^) expressing the activation/inhibition surface markers are presented. PHA (5 μg/mL) were used as control for the expression of surface markers. ^∗^*P* < 0.05, ^∗∗^*P* < 0.01, and ^∗∗∗^*P* < 0.001. ^∗^Significant differences were observed in comparison with unstimulated cells (two-way ANOVA with Bonferroni post-test).

Supplementary Figure 5 Analysis of immunomodulatory effects on expression of activation surface markers in PBMC stimulated with OMVs from *B. melitensis*. PBMCs from healthy donor were stimulated with 25 μg/mL of OMVs from *B. melitensis* 16M and VTRM1 or PHA only (5 μg/mL). Cells were pre-incubated with OMVs, and then re-stimulated with 5 μg of PHA to assess the inhibitory effect of the OMVs in the expression of surface markers. Activation was measured by flow cytometry using mAbs against surface molecules CD69 and CD86. Expression was measured by mean fluorescence intensity (MFI) of each surface marker from the gated cells: monocytes (CD91^+^) and lymphocytes (CD3^+^, CD19^+^) and the percentage of gated cells also is shown. The gated CD19^+^CD69^+^ **(A,B)**, CD91^+^CD69^+^ **(C,D)**, CD3^+^CD86^+^ **(F,G)**, CD19^+^CD86^+^ **(H,I)**, and CD91^+^CD86^+^ **(J,K)** cells did not change the expression or percentage of gated cells expressing activation surface markers.

Supplementary Figure 6 Analysis of immunomodulatory effect on expression of inhibition surface markers in PBMC stimulated with OMVs from *B. melitensis*. PBMCs from healthy donor were stimulated with 25 μg/mL of OMVs from *B. melitensis* 16M and VTRM1 or PHA (5 μg/mL). Cells pre-incubated with OMVs were re-stimulated with 5 μg of PHA to assess the inhibitory effect of OMVs in the expression of surface markers. Activation was measured by flow cytometry using mAbs against surface molecules PD-1 and PD-L1. Expression was measured with the Mean fluorescence intensity (MFI) of each surface marker from the gated cells: monocytes (CD91^+^) and lymphocytes (CD3^+^, CD19^+^) and the percentage of gated cells also is shown. The gated CD3^+^PD-1^+^ **(A,B)**, CD19^+^PD-L1^+^ **(C,D)**, CD91^+^PD-1^+^ **(E,F)**, and CD91^+^PD-L1^+^ **(G,H)** cells did not change the expression or percentage of gated cells expressing inhibition surface markers.

Supplementary Figure 7 Morphological changes induced by OMVs from *B. melitensis* in PBMCs. After 2 h post-stimulation with OMVs from smooth and rough *B. melitensis* strains morphological changes of PBMCs were observed compared with unstimulated PBMCs. Cells stimulated with OMVs showed differences in the affinity of the dye to the nucleus (DAPI) displaying enlarged shaped. Confocal microscopy did not show evident defective actin **(A)** and tubulin **(B)** polymerization in PBMCs stimulated with OMVs from both *Brucella* strains. Bars = 10 μm.

Supplementary Table 1 Identified proteins in OMVs from *B. melitensis* 16M and VTRM1.

Supplementary Table 2 Identified proteins in OMVs from *B. melitensis* observed in the electrophoretic profile at 20 and 23 kDa.
