# Supplementary figures and images for: Outer Membrane Vesicles From Brucella melitensis Modulate Immune Response and Induce Cytoskeleton Rearrangement in Peripheral Blood Mononuclear Cells
Source: Front Microbiol. 2020 Oct 19;11:556795. doi: 10.3389/fmicb.2020.556795 (PMC7604303; doi:10.3389/fmicb.2020.556795)

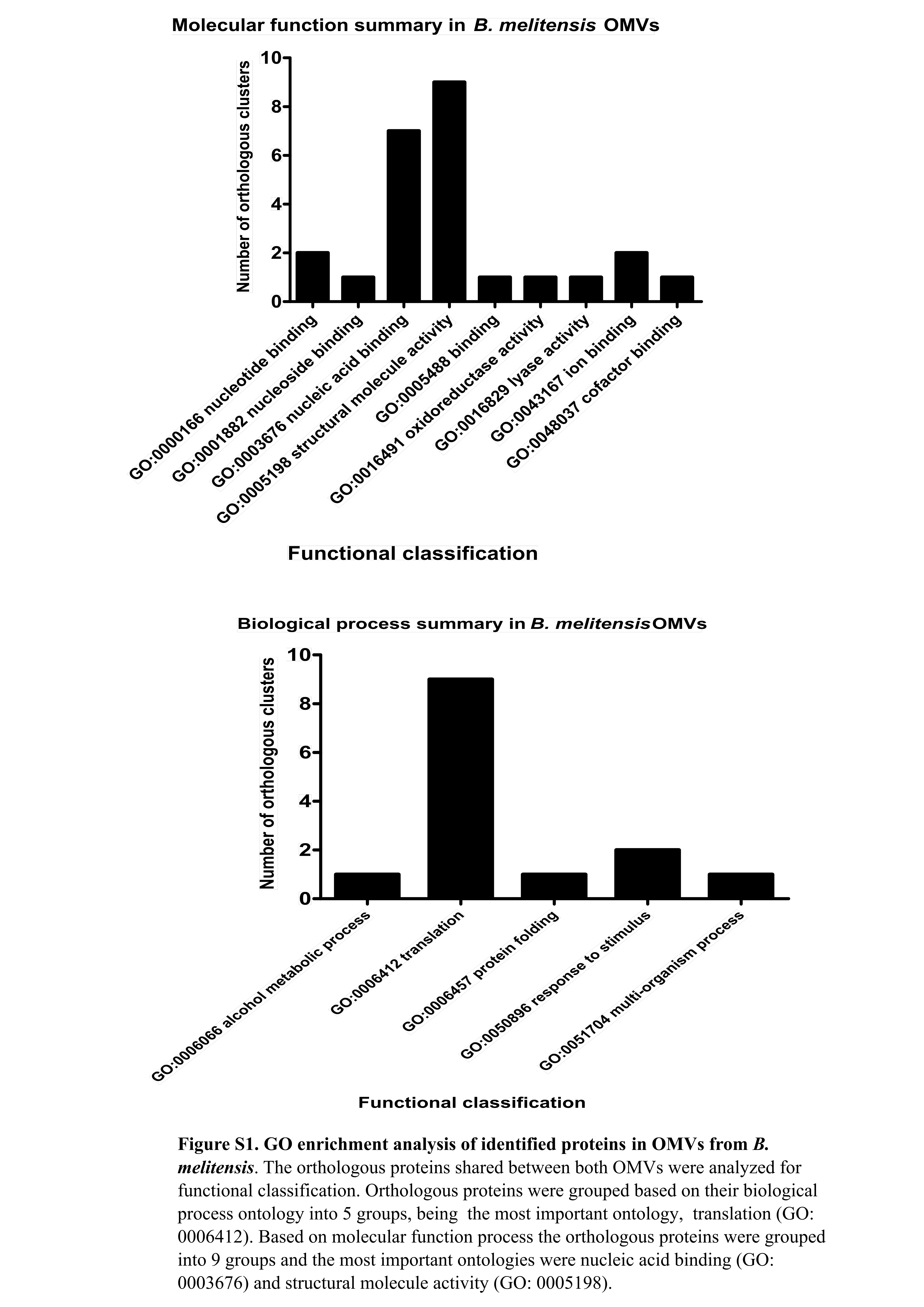

Supplement: Supplementary Figure 1 — GO enrichment analysis of identified proteins in OMVs from B. melitensis. The orthologous proteins shared between both OMVs were analyzed for functional classification. Orthologous proteins were grouped based on their biological process ontology into five groups, being the most important ontology, translation (GO: 0006412). Based on molecular function process the orthologous proteins were grouped into nine groups and the most important ontologies were nucleic acid binding (GO: 0003676) and structural molecule activity (GO: 0005198). [file Image_1.TIF]

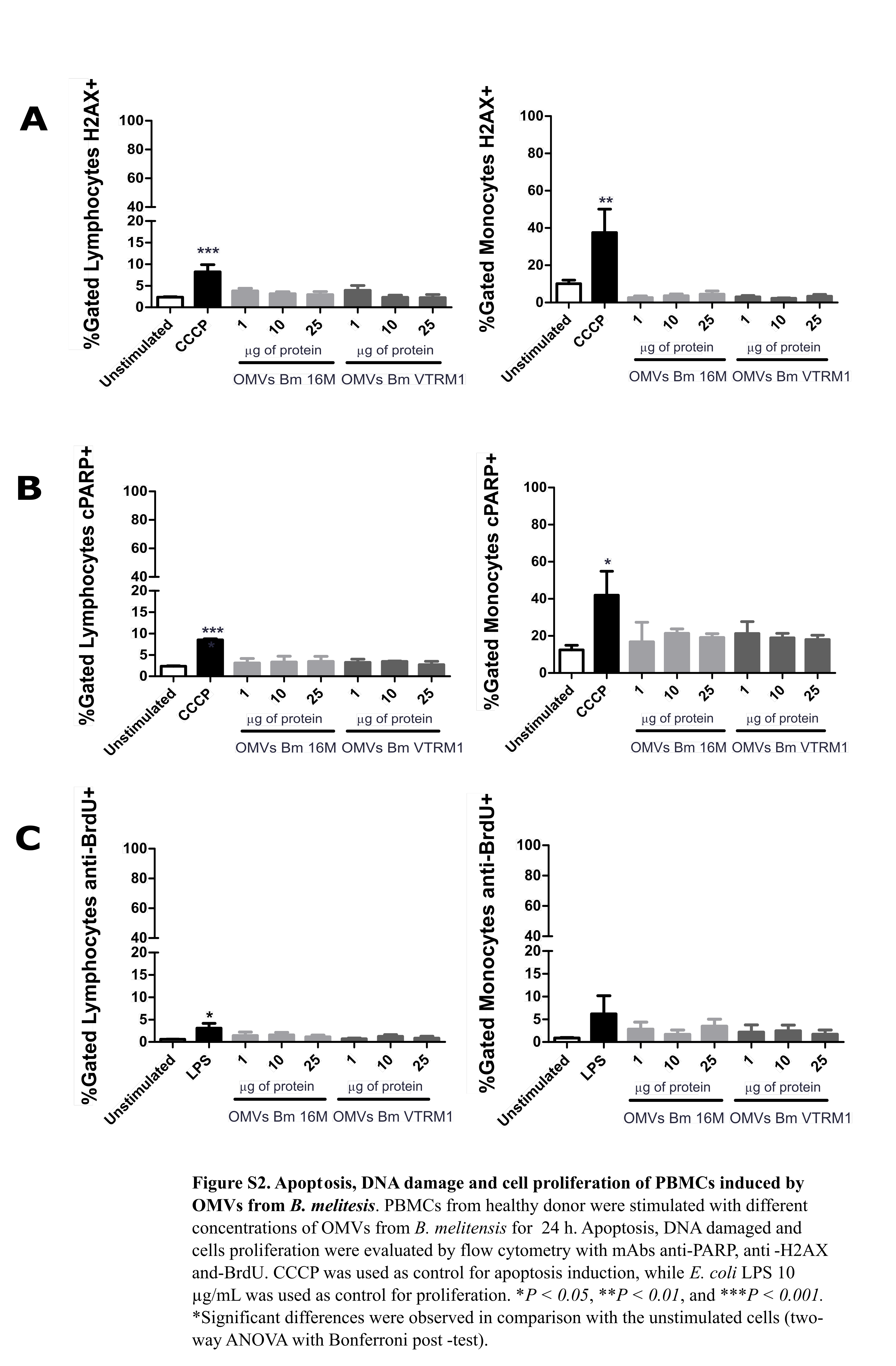

Supplement: Supplementary Figure 2 — Apoptosis, DNA damage and cell proliferation of PBMCs induced by OMVs from B. melitesis. PBMCs from healthy donor were stimulated with different concentrations of OMVs from B. melitensis for 24 h. Apoptosis, DNA damaged and cells proliferation were evaluated by flow cytometry with mAbs anti-PARP, anti-H2AX and-BrdU. CCCP was used as control for apoptosis induction, while E. coli LPS 10 μg/mL was used as control for proliferation. ∗P < 0.05, ∗∗P < 0.01, and ∗∗∗P < 0.001. ∗Significant differences were observed in comparison with the unstimulated cells (two-way ANOVA with Bonferroni post-test). [file Image_2.TIF]

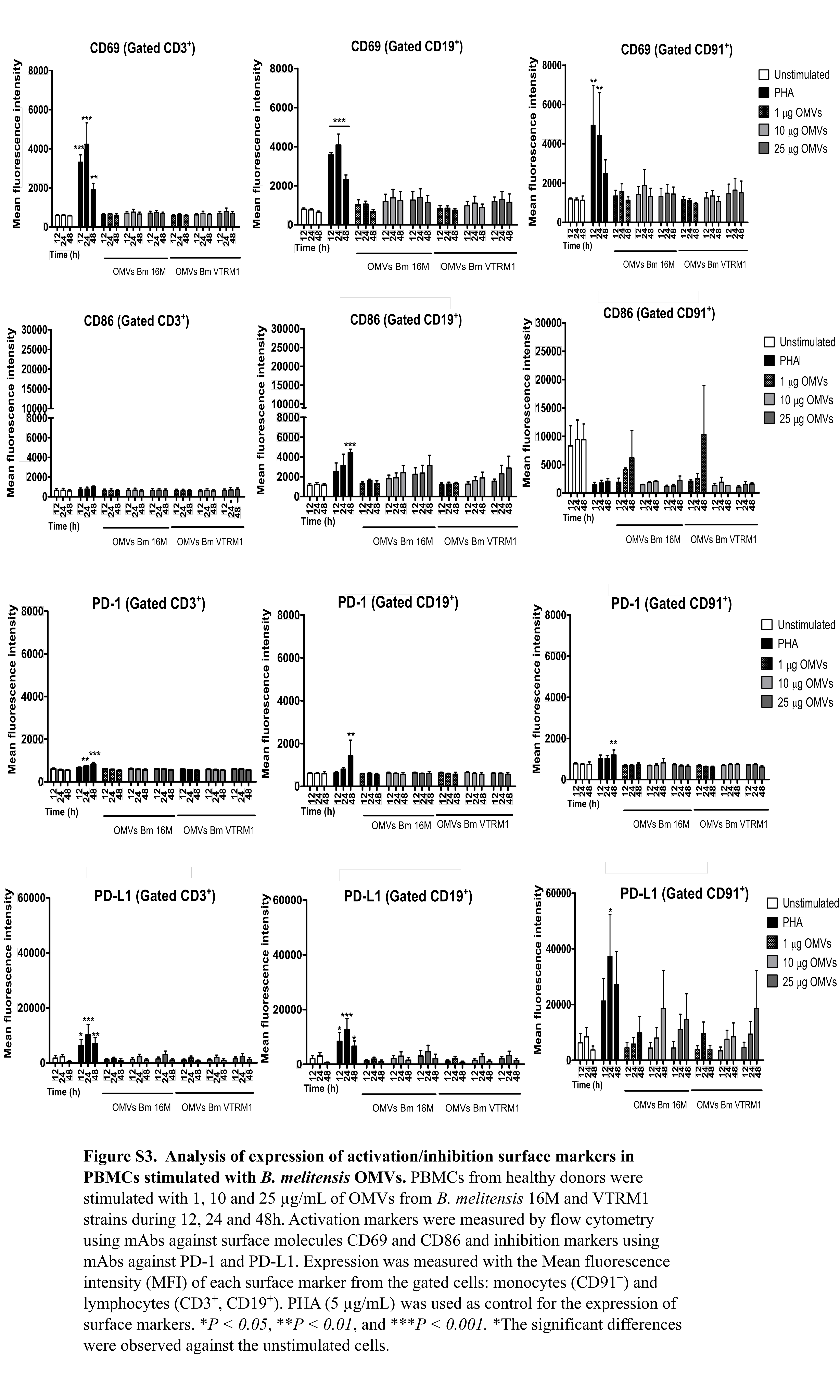

Supplement: Supplementary Figure 3 — Analysis of expression of activation/inhibition surface markers in PBMCs stimulated with B. melitensis OMVs. PBMCs from healthy donors were stimulated with 1, 10, and 25 μg/mL of OMVs from B. melitensis 16M and VTRM1 strains during 12, 24, and 48 h. Activation markers were measured by flow cytometry using mAbs against surface molecules CD69 and CD86 and inhibition markers using mAbs against PD-1 and PD-L1. Expression was measured with the Mean fluorescence intensity (MFI) of each surface marker from the gated cells: monocytes (CD91+) and lymphocytes (CD3+, CD19+). PHA (5 μg/mL) was used as control for the expression of surface markers. ∗P < 0.05, ∗∗P < 0.01, and ∗∗∗P < 0.001. ∗The significant differences were observed against the unstimulated cells. [file Image_3.TIF]

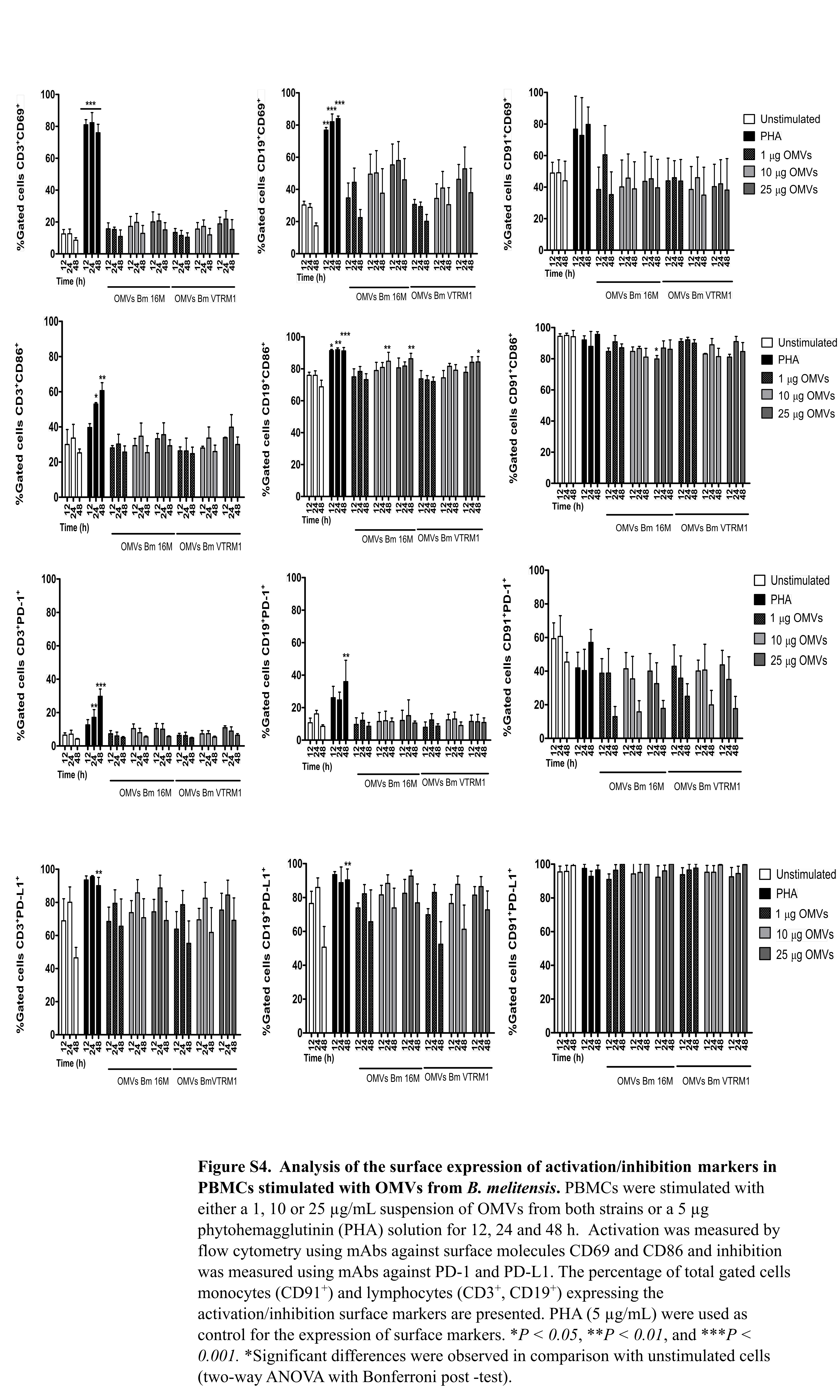

Supplement: Supplementary Figure 4 — Analysis of the surface expression of activation/inhibition markers in PBMCs stimulated with OMVs from B. melitensis. PBMCs were stimulated with either a 1, 10, or 25 μg/mL suspension of OMVs from both strains or a 5 μg phytohemagglutinin (PHA) solution for 12, 24, and 48 h. Activation was measured by flow cytometry using mAbs against surface molecules CD69 and CD86 and inhibition was measured using mAbs against PD-1 and PD-L1. The percentage of total gated cells monocytes (CD91+) and lymphocytes (CD3+, CD19+) expressing the activation/inhibition surface markers are presented. PHA (5 μg/mL) were used as control for the expression of surface markers. ∗P < 0.05, ∗∗P < 0.01, and ∗∗∗P < 0.001. ∗Significant differences were observed in comparison with unstimulated cells (two-way ANOVA with Bonferroni post-test). [file Image_4.TIF]

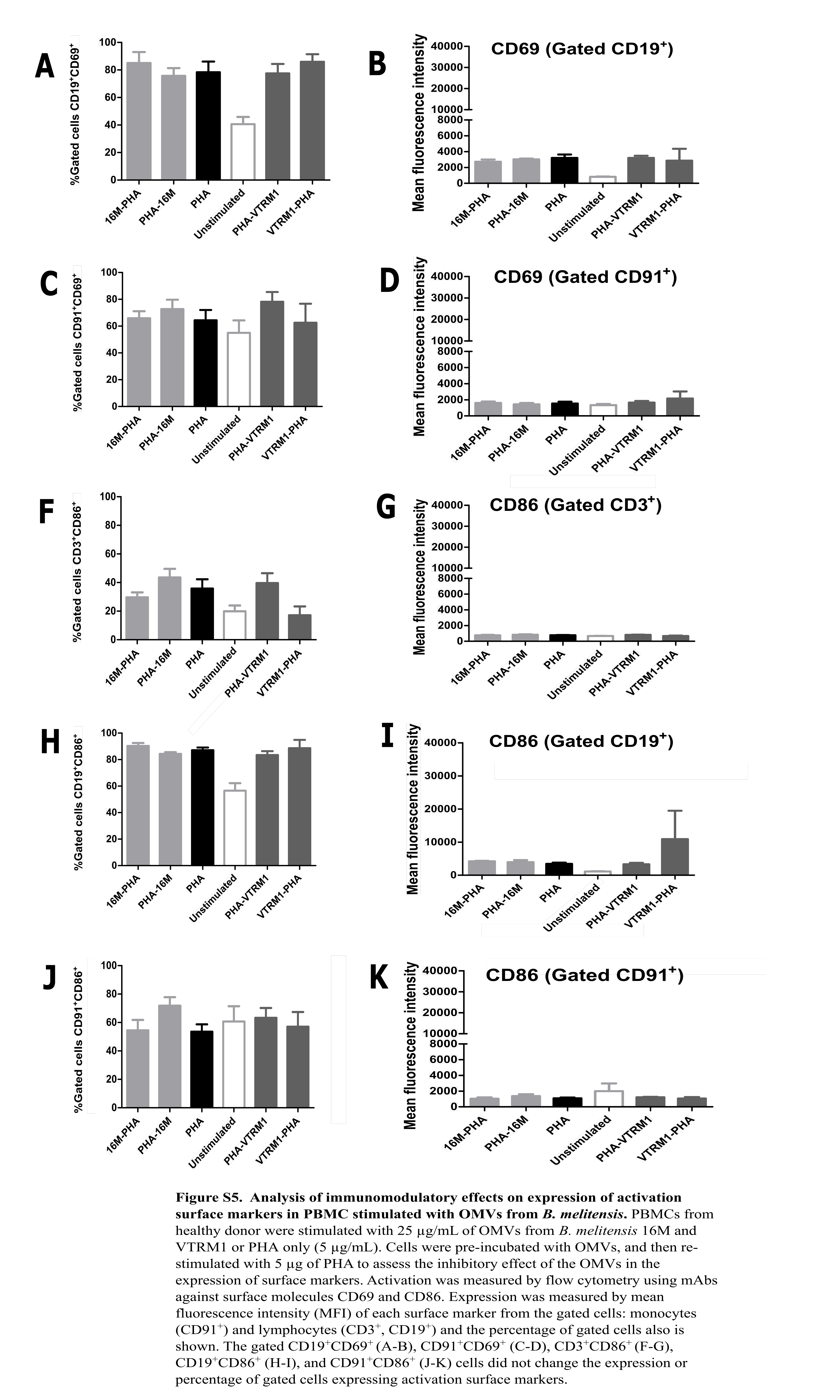

Supplement: Supplementary Figure 5 — Analysis of immunomodulatory effects on expression of activation surface markers in PBMC stimulated with OMVs from B. melitensis. PBMCs from healthy donor were stimulated with 25 μg/mL of OMVs from B. melitensis 16M and VTRM1 or PHA only (5 μg/mL). Cells were pre-incubated with OMVs, and then re-stimulated with 5 μg of PHA to assess the inhibitory effect of the OMVs in the expression of surface markers. Activation was measured by flow cytometry using mAbs against surface molecules CD69 and CD86. Expression was measured by mean fluorescence intensity (MFI) of each surface marker from the gated cells: monocytes (CD91+) and lymphocytes (CD3+, CD19+) and the percentage of gated cells also is shown. The gated CD19+CD69+ (A,B), CD91+CD69+ (C,D), CD3+CD86+ (F,G), CD19+CD86+ (H,I), and CD91+CD86+ (J,K) cells did not change the expression or percentage of gated cells expressing activation surface markers. [file Image_5.TIF]

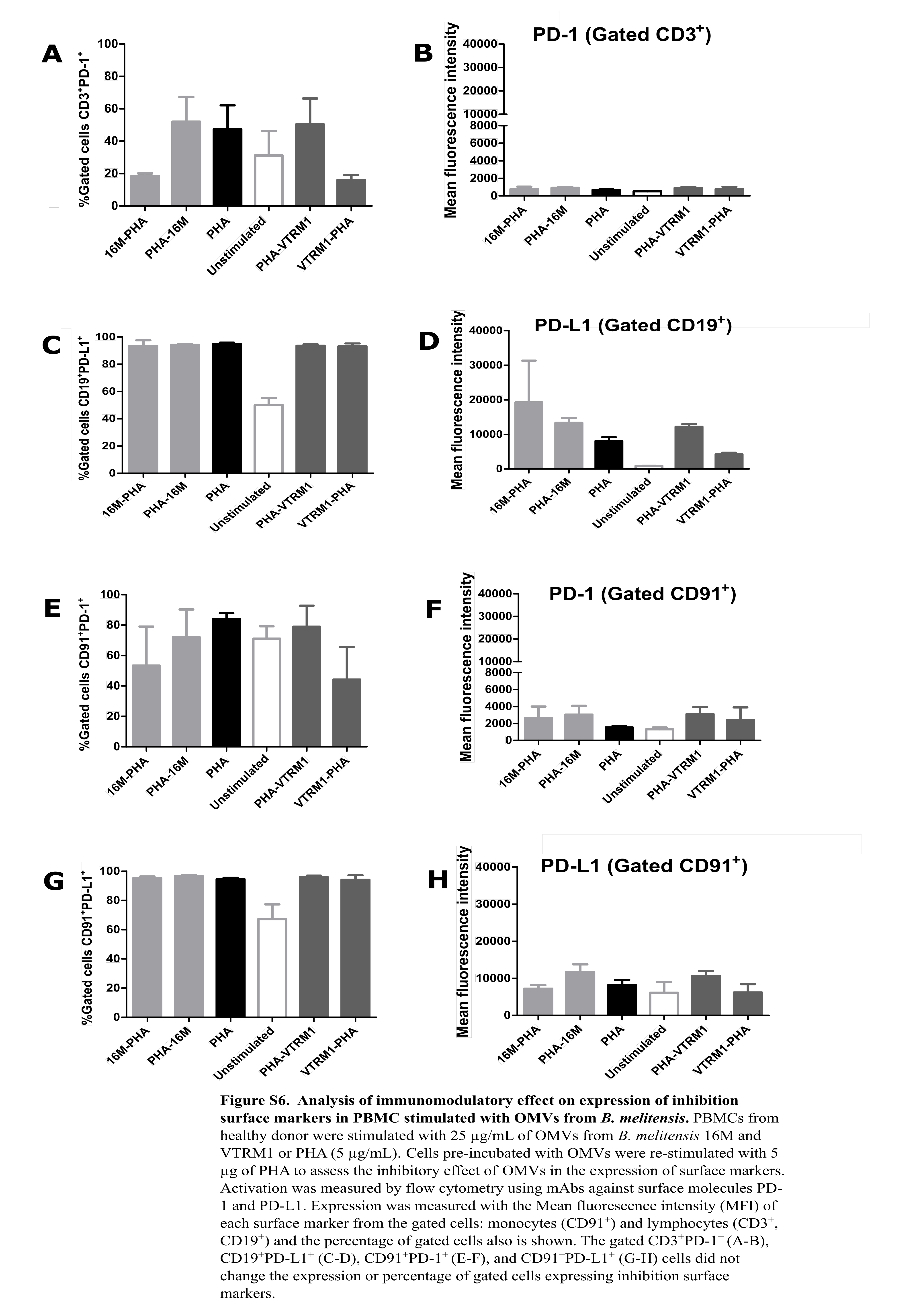

Supplement: Supplementary Figure 6 — Analysis of immunomodulatory effect on expression of inhibition surface markers in PBMC stimulated with OMVs from B. melitensis. PBMCs from healthy donor were stimulated with 25 μg/mL of OMVs from B. melitensis 16M and VTRM1 or PHA (5 μg/mL). Cells pre-incubated with OMVs were re-stimulated with 5 μg of PHA to assess the inhibitory effect of OMVs in the expression of surface markers. Activation was measured by flow cytometry using mAbs against surface molecules PD-1 and PD-L1. Expression was measured with the Mean fluorescence intensity (MFI) of each surface marker from the gated cells: monocytes (CD91+) and lymphocytes (CD3+, CD19+) and the percentage of gated cells also is shown. The gated CD3+PD-1+ (A,B), CD19+PD-L1+ (C,D), CD91+PD-1+ (E,F), and CD91+PD-L1+ (G,H) cells did not change the expression or percentage of gated cells expressing inhibition surface markers. [file Image_6.TIF]

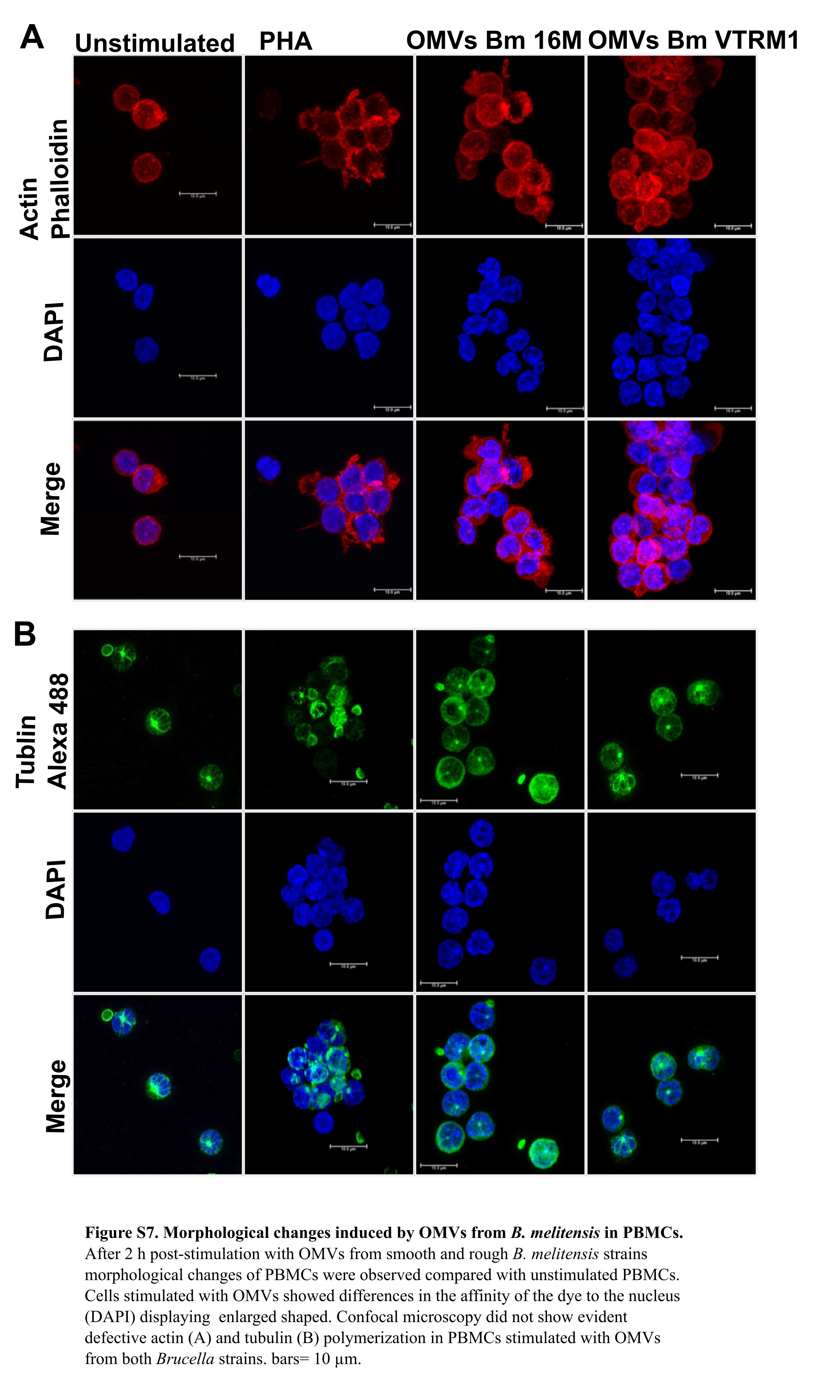

Supplement: Supplementary Figure 7 — Morphological changes induced by OMVs from B. melitensis in PBMCs. After 2 h post-stimulation with OMVs from smooth and rough B. melitensis strains morphological changes of PBMCs were observed compared with unstimulated PBMCs. Cells stimulated with OMVs showed differences in the affinity of the dye to the nucleus (DAPI) displaying enlarged shaped. Confocal microscopy did not show evident defective actin (A) and tubulin (B) polymerization in PBMCs stimulated with OMVs from both Brucella strains. Bars = 10 μm. [file Image_7.TIF]
